# Evaluating the Microbubble Hypothesis: Apparent Air−Water CH_4_ and CO_2_ Gas Transfer Velocities in Relation to Microbubble Concentrations in a Meso-Oligotrophic Lake

**DOI:** 10.1021/acs.est.6c05689

**Published:** 2026-09-07

**Authors:** Mohammad Atif Khan, Philipp S. Keller, Georgiy Kirillin, Hans-Peter Grossart, Stella A. Berger, Martin Lienenlüke, Jens C. Nejstgaard, Mats Öquist, Marcus Klaus

**Affiliations:** † Department of Forest Ecology and Management, Swedish University of Agricultural Sciences, Skogsmarksgränd 17, 90183 Umeå, Sweden; ‡ Department of Plankton and Microbial Ecology, Leibniz Institute of Freshwater Ecology and Inland Fisheries (IGB), 16775 Stechlin, Germany; § Institute of Biochemistry and Biology, Potsdam University, 14469 Potsdam, Germany; ∥ Department of Ecohydrology and Biogeochemistry, Leibniz Institute of Freshwater Ecology and Inland Fisheries (IGB), 12587 Berlin, Germany

**Keywords:** microbubble void fraction, surface microlayer, air−water gas exchange, piston velocity, greenhouse gas, inland water

## Abstract

Aquatic systems exchange large amounts of carbon dioxide (CO_2_) and methane (CH_4_) with the atmosphere. The microbubble hypothesis suggests that microbubbles preferentially enhance air−water transfer of sparingly soluble gases like CH_4_ over CO_2_, explaining elevated normalized gas transfer velocity ratios (*k*_600_,_CH4_ *:k*_600_,_CO2_) observed in many inland waters. This hypothesis remains to be empirically tested. We tested this hypothesis in meso-oligotrophic Lake Stechlin (NE Germany) using acoustic bubble spectroscopy to quantify microbubble concentrations, floating chambers to measure CH_4_ and CO_2_ fluxes, and dissolved gas concentrations to estimate *k*_600_ for CH_4_ and CO_2_. Contrary to our hypothesis, *k*_600_,_CH4_ *:k*_600_,_CO2_ averaged near unity and was independent of microbubble concentration. Theoretical microbubble-mediated CH_4_ fluxes contributed ∼0.05% to total CH_4_ fluxes. Thus, microbubbles affected gas transfer velocities weakly under the relatively low-wind, posteutrophication conditions of our sampling campaign. Instead, *k*_600_,_CH4_ *:k*_600_,_CO2_ was more likely explained by gas-specific near-surface concentration gradients, surfactant-induced surface immobilization, and chemical enhancement of CO_2_ exchange. Microbubble concentrations were mainly linked to wind-induced turbulence and were elevated in littoral areas. Our results limit the applicability of the microbubble hypothesis and suggest that larger microbubble gas fluxes likely require stronger wind-induced forcing than observed here, or site-specific sources, such as littoral vegetation.

## Graphical abstract

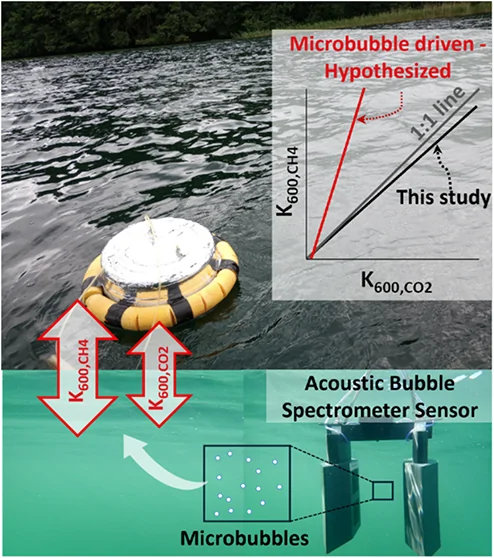

## Introduction

Inland waters, including lakes, are significant sources of carbon dioxide (CO_2_) and methane (CH_4_) to the atmosphere,^1–4^ thus impacting the global carbon cycle and climate. Globally, inland waters collectively emit an estimated 5.55 Pg CO_2_ yr^−1^ and 100.3 Tg CH_4_ yr^−1^ to the atmosphere, with lakes contributing 0.70 Pg CO_2_ yr^−1^ and 59.6 Tg CH_4_ yr^−1^ to these fluxes.^5,6^ However, these flux estimates carry large uncertainties of roughly 30−80%, partly owing to the limited knowledge and ability to adequately resolve the mechanisms influencing gas exchange rates.^7,8^ Solid mechanistic understanding of the processes that govern air−water gas transfer is critical not only for accurate carbon budgeting but also for predicting responses to changing environmental conditions.^6^

In lakes, diffusion constitutes the main pathway of CO_2_ emission and an important pathway for CH_4_ exchange. The diffusive flux is determined by the product of the gas concentration gradient across the air−water interface and the air−water gas transfer velocity (*k*). In lakes, *k* can be estimated using air−water flux measurements by floating chambers, tracer gas experiments, or eddy covariance and gas concentration measurements, or modeled using wind speed^9^ or convection.^10^ The *k* is primarily controlled by near-surface water-side turbulence, but also by other properties such as surface films (surfactants).^11^ To enable standardization across different gases and temperatures, *k* is commonly normalized through Schmidt-number (*Sc*) scaling, yielding *k*_600_, which represents *k* adjusted to *Sc* = 600 (corresponding to CO_2_ in freshwater at 20 °C).^12^ This approach implies that *k*_600_ for different gases should be identical if gas exchange occurs solely by diffusion under the same environmental conditions. However, this idealized assumption may not hold true under natural conditions.

Growing evidence suggests that apparent *k*_600_ differs for CO_2_ and CH_4_, with *k*_600_ ratios of CH_4_ over CO_2_ ranging from 0.36 to 2.5 across various freshwater systems.^13–19^ The observed discrepancy in apparent *k*_600_ for CO_2_ and CH_4_ may reflect the net effect of several, potentially opposing gas-specific biogeochemical production or consumption processes in the surface microlayer, which alter vertical CH_4_ and CO_2_ concentration gradients and may be captured differently by different estimation methods.^17^ For example, higher *k*_600_ ratios of CH_4_ over CO_2_ could be explained by oxic CH_4_ production.^20^ Conversely, lower *k*_600_ ratios of CH_4_ over CO_2_ were attributed to microbial respiration and chemical enhancement of CO_2_ exchange through carbonate buffering reactions, especially under higher pH and low turbulent conditions.^17^ A *k*_600_ ratio of CH_4_ over CO_2_ larger than unity has also been attributed to the presence of microbubbles (<100 μm diameter) in estuaries,^14^ rivers,^18^ lakes, reservoirs,^15,19^ wetlands^21^ and coastal systems.^22^ Microbubbles strip CH_4_ more effectively than CO_2_, owing to its lower solubility. If sufficient and long-lived, microbubbles can therefore preferentially carry low solubility gases to the air−water interface, leading to apparent *k*_600_ values that exceed those for more soluble gases. Despite being formulated more than a decade ago, the microbubble hypothesis remains, to the best of our knowledge, to be empirically tested.

The presence of microbubbles in aquatic systems was first reported in the 1950s for the open ocean,^23^ and their evolution mechanism and distribution have been studied extensively ever since.^24–26^ In the ocean, wave breaking entrains bubbles across a broad size spectrum, with microbubbles following a typical −3/2 power-law scaling, and being stabilized by natural surfactants that reduce surface tension and inhibit gas dissolution.^27^ Bubbles contribute to air−sea gas exchange in the oceans, with bubble size as a key controlling factor, although the magnitude of the microbubble contribution to total gas transfer remains uncertain.^28^ In freshwaters, direct observations of microbubbles remain scarce. The few existing studies indicate that microbubble concentrations in freshwater lakes can be of similar magnitude as those in marine environments under comparable hydrodynamic conditions.^29,30^ Microbubble formation in lakes may result from physical processes (wind-driven surface turbulence,^15^ wave breaking, rainfall impact,^31^ boat traffic,^32^ crevice-type nucleation^33^ on rough surfaces), and biological processes (photosynthesis by aquatic primary producers,^34,35^ excretion in zooplankton,^36^ and oxic methane production^20^). These factors are expected to vary spatially and temporally within lakes under shifting environmental conditions. Yet, their potentially diverse effects on *k*_600_ ratios of CH_4_ over CO_2_ remain largely unexplored.

The central empirical question behind the microbubble hypothesis is whether microbubble abundance is sufficient to enhance air−water transfer for CH_4_ (relative to CO_2_) under typical lake conditions.^15,19^ We therefore evaluate the microbubble hypothesis through coupled estimates of *k*_600_ for CH_4_ and CO_2_ and microbubble concentrations in Lake Stechlin, a meso-oligotrophic freshwater lake in northeastern Germany. This lake provides an ideal setting due to previous investigations highlighting the significant enhancement of CH_4_ transfer relative to CO_2_, supporting the microbubble hypothesis under wind-driven surface turbulence conditions.^15^

Our main objectives were to evaluate how microbubbles influence apparent *k*_600_ ratios of CH_4_ over CO_2_ (*k*_600,CH4_ *:k*_600,CO2_) (**O1**) and to identify drivers of microbubble concentrations (**O2**) under diverse environmental conditions across Lake Stechlin. We hypothesized that microbubbles enhance *k*_600,CH4_ *:k*_600,CO2_ (**H1**), implying that the following conditions would collectively be fulfilled: *k*_600,CH4_ *:k*_600,CO2_ is >1 (**H1a**) and increases with microbubble concentrations (**H1b**), and microbubbles contribute significantly to the total CH_4_ flux (**H1c**). We also hypothesized that microbubble concentrations are increasing with wind-driven turbulence and particle abundance and are higher in littoral areas, especially with abundant macrophyte coverage (**H2**). In case H1 was rejected, we also aimed to explore alternative drivers for spatiotemporal variability in *k*_600,CH4_ *:k*_600,CO2_, including wind speed, turbulence, and chemical enhancement of CO_2_ exchange (**O3**).

## Material and Methods

### Description of the Study Site

The field campaign was conducted in Lake Stechlin, northeastern Germany (53°09′N, 13°02′E, Figure S1), from June 11 to 19, 2024. Lake Stechlin is a dimictic lake that underwent a recent shift from oligotrophic to eutrophic conditions^37^ and, in the past five years, shifted to meso-oligotrophic conditions. The lake covers an area of 4.23 km^2^, with a mean depth of 23.3 m. We sampled an offshore midlake site at 15 m depth (M), the deepest point at 69.5 m (D), and two littoral transects: one macrophyte-rich, reed-dominated (R) and one without reed and sparse macrophyte cover (N) (Figure S2). Along each transect, four sites (R1−R4 and N1−N4) were sampled at ∼5−50 m distance from shore, covering 0.3, 0.75, 1.5, and 5 m water depth, respectively. The M site was sampled on 16 occasions to capture varying weather and hydrodynamic conditions, whereas D and all nearshore sites were sampled two times on different days to primarily capture spatial variability. To limit daytime-related bias, the sampling order of the R and N transects was reversed during the second sampling day.

### Environmental and Meteorological Observations

Surface water temperature, electrical conductivity, pH, and air pressure were measured with field-calibrated probes (GMH 5500, GHM Messtechnik GmbH, Standort Greisinger, Regenstauf, Germany, WA-100ATC Pure Water tester, Voltcraft, Conrad Electronic SE, Hirschau, Germany, and ProODO, YSI Inc., Yellow Springs, OH). Wind speed was measured at 3 m height by a floating eddy-covariance tower (equipped with a Gill WindMaster Pro ultrasonic anemometer), located close to site M (https://www.igb-berlin.de/en/infrastructure/lakelab), and normalized to 10 m height.^38^

### Microbubble Measurements: Acoustic Bubble Spectrometry (ABS)

We quantified microbubble size distributions and void fractions (gas volume per unit water volume) using a field-deployable Weather Resistant Acoustic Bubble Spectrometer® (Dynaflow Inc., Jessup, MD), which infers bubble properties from their effects on acoustic wave propagation. The system employs paired 5 × 5 cm^2^ hydrophones operated at 250 kHz, mounted in streamlined PVC holders at a fixed transmitter−receiver distance of 7.5 cm and controlled via a PC-based DAQ system (Figure S3). Prior to measurements at each site at around 5 cm depth, we recorded reference signals in degassed lake water at *in situ* temperature for calibration purposes. Bubble size and void fraction distributions were retrieved for a series of radius classes (*R* = 8.94−14.82, 14.82−20.7, 20.7−26.58, 26.58−32.46, 32.46−38.34, 38.34−44.22 μm) from frequency-dependent sound speed and attenuation between 60 and 500 kHz using standard inversion and quality control procedures (details in Supporting Methods Text S1).

### Air−Water Flux Measurements

Air−water CO_2_ and CH_4_ fluxes were measured using two floating chambers (Figure S4) coupled to a portable CH_4_/CO_2_ analyzer (LI-7810, LI-COR, Lincoln, NE; 250 sccm). The first chamber, a dome-shaped “flying” chamber (6.4 L; 0.047 m^2^) with a thin skirt, was suspended from a buoyant “spider-like” frame with flexible coupling to minimize surface disturbance, floating on the water on buoys attached to each of its legs.^14^ The second chamber was a conventional flat-topped floating chamber (12.15 L; 0.126 m^2^) with a foam collar. Both chambers were reflective-coated and connected via separate inlet/outlet lines to the analyzer. At each site, three replicate deployments (≥6 min each) per chamber were conducted alternately with the boat anchored. The flying chamber was used to test potential overestimation from an anchored floating chamber due to artificial turbulence.^39^ Comparisons (Figures S8, S10) showed the flat-topped floating chamber produced more consistent *k*_600_ estimates and is hence reported here (unless stated otherwise), enabling comparison with previous studies, including Lake Stechlin.^15^

CO_2_ and CH_4_ fluxes were calculated using the *R*-package “FluxCalR”:^40^

1 $$F=\frac{V}{R\times T\times A}\times \frac{\Delta c}{\Delta t}\times \rho$$

where *V* is chamber volume (L) (including tubing), *R* is the gas constant (L atm mol^−1^ K^−1^), *T* is the temperature (K), *A* is the chamber area (m^2^), *Δc*/Δ*t* is the rate of change in gas partial pressure (s^−1^), and ρ is the air pressure (atm). For short-term deployments, concentration changes were linear; Δ*c*/Δ*t* was the slope of the least-squares regression with the highest *R* ^2^ among all regressions over ∼4 min within a 6 min deployment window, excluding the initial 30 s, and excluding deployments showing nonlinear concentration spikes induced by larger bubbles. Mean ± SD *R* ^2^ values were 0.99 ± 0.01 (CH_4_) and 0.93 ± 0.09 (CO_2_) for the flying chamber, and 0.98 ± 0.03 (CH_4_) and 0.92 ± 0.13 (CO_2_) for the floating chamber.

### Dissolved CH_4_ and CO_2_ Measurements

We quantified dissolved CH_4_ and CO_2_ concentrations in near-surface water through analysis of headspace air of 5 mL water samples using a gas chromatograph (GC: Clarus 580 with a Turbomatrix 110 Headspace autosampler, PerkinElmer, Shelton, CT). We verified these measurements by two independent methods: deploying a portable greenhouse gas analyzer, one based on onsite continuous measurements using a gas equilibrator and the other based on a manual headspace method. For method details and intercomparisons, see Supporting Text S2. The data shown in the remainder of this paper are based on the GC method.

### Calculation of Gas Transfer Velocities

The gas transfer velocities of CO_2_ and CH_4_ were derived from concomitant measurements of gas fluxes (*F*) and partial pressure differences in water and the atmosphere using Fickian diffusion theory

2 $$k=\frac{F}{{K_{H}(P}_{w}-P_{a})}$$

where *P*_w_ and *P*_a_ are the gas partial pressures in near-surface water and the atmosphere, respectively, and *K*_H_ is Henry’s solubility constant, calculated separately for CO_2_ and CH_4_ using surface water temperature: directly from the Weiss formula for CO_2_,^41^ and for CH_4_ by converting Bunsen solubility^41^ to Ostwald solubility^42^ and subsequently to Henry solubility.^43^ To facilitate comparison across environmental conditions and gases, *k* was normalized to *Sc* = 600 as a function of temperature^41^

3 $$k_{600}=k\times {\left(\frac{600}{\mathrm{Sc}}\right)}^{-n}$$

where *n* = 2/3 for wind speed <3.7 m s^−1^ and *n* = 1/2 for wind speed ≥3.7 m s^−1^.^44^

### Chemical Enhancement of CO_2_ Exchange

Theoretical chemical enhancement of CO_2_ exchange due to carbonate equilibrium reactions was calculated as^45,46^

4 $$\alpha =\frac{T}{\left(T-1\right)+\frac{\tan h\left(\mathrm{Qz}\right)}{\mathrm{Qz}}}$$

where *T* = 1+*a*_H_^2^(*K*’_1_ *K*’_2_+*K*’_1_ *a*_H_)^−1^ is the carbonate speciation factor, *a*_H_ = 10^−pH^ is the hydrogen ion activity, *K*′_1_ and *K*′_2_ are the carbonate equilibrium constants,^47^ *Q* = (*rT*/*D*)^1/2^, *D* is the molecular diffusivity of CO_2_,^48^ *r* = *r*_1_+*r*_2_ *K*′_w_ *a*_H_^−1^ is an effective first-order rate constant, *r*_1_ and *r*_2_ are the rate constants for CO_2_ hydration by water and OH^−^,^49^ *K*′_w_ is the apparent dissociation constant of water,^50^ *z* = *D*/*k*_u_ is the surface boundary layer thickness, and *k*_u_ is the unenhanced measured normalized gas transfer velocity of CO_2_. For each calculation, *in situ* temperature, electrical conductivity, and pH were used to calculate salinity^51^ and water density,^52^ and all were used as inputs to the above parametrizations. As α was estimated from water properties measured at 5 cm water depth, unresolved microscale gradients within the upper few centimeters of the water column may contribute to uncertainty in the α-based interpretation.

### Microbubble CH_4_ Flux Calculations

Theoretical maximum microbubble CH_4_ fluxes were estimated by combining ABS-derived void fraction with a model of bubble rise velocity and gas exchange. For each bubble radius class, the bin midpoint was used as the effective radius, and the microbubble rise velocity (w_b_) was computed from bubble radius^53^ assuming dirty bubbles, i.e., bubbles coated with surface-active substances that behave hydrodynamically more like rigid particles than clean bubbles.^53^ CH_4_ in the microbubbles was assumed to be in equilibrium with dissolved CH_4_, with partial pressure given by Henry’s law: pCH_4_ = *C*_w,CH4_/K_H,CH4_, where *C*_w,CH4_ is the water CH_4_ concentration. The CH_4_ mole fraction in the bubble (x_CH4_) was calculated by dividing pCH_4_ by the total pressure inside the bubble, defined as the sum of atmospheric pressure, hydrostatic pressure at the bubble depth, and Laplace overpressure (2σ/*R*, where σ is the surface tension^54^ and *R* is the bubble radius). Molar gas density inside the bubble (*n*/*V*) was calculated via the ideal gas law. For each radius class *i*, the upward microbubble CH_4_ flux was calculated as *F_i_* = v_i_ w_b,i_ x_CH4_ *n*/*V*, where v is the void fraction. This yields an areal CH_4_ flux (mmol m^−2^ d^−1^) per radius class and was finally summed over all radius classes to derive the total microbubble CH_4_ flux ∑*_i_* *F_i_*.

This approach assumes that microbubble-internal CH_4_ concentrations equilibrate with the surrounding water before the microbubbles, stabilized by surfactants, rise to the surface, and are solely driven by buoyancy. This assumption is supported by a bubble gas-exchange model (Supporting Text S5; Figure S14), which showed that bubbles equilibrate within seconds, likely faster than their rise to the surface. We consider our estimate an upper limit, if microbubbles are formed through surface entrainment or photosynthesis and may not fully equilibrate if entrained sufficiently shortly,^55^ or if they dissolve before surfacing. We acknowledge that fluxes may be higher if microbubbles were formed through methanogenesis at nucleation sites because these may initially contain pure CH_4_. This pathway remains a potential mechanism, although we lack empirical evidence to assess its relevance here empirically.

### Turbulent Kinetic Energy Dissipation

At the midlake site (M), near-surface turbulence was quantified as turbulent kinetic energy (TKE) dissipation rates (ε) from current velocities using a 5-beam 1 MHz Acoustic Doppler Current Profiler (Signature1000 AD2CP, Nortek AS, Norway). It was deployed upward-looking at 15 m depth and recorded velocities from 3.42−0.68 m below the surface (0.02 m resolution) through hourly 10 min bursts (2 Hz) using pulse-coherent high-resolution mode. TKE dissipation rates were computed using the velocity structure function method,^56^ applied to the along-beam velocity fluctuations on the vertical beam with inertial-subrange fitting and noise correction. Three maximum separation ranges (*r* = 0.4, 0.5, 0.6 m) yielded similar results (<10% difference); *r* = 0.4 m was selected due to the lowest fraction of rejected values. Analyses used ε from the uppermost layer (0.68−1.08 m). Full details are provided in Supporting Text S3.

At nearshore sites (R1−R3, N1−N3), flow velocities were measured using an Acoustic Doppler Velocity meter (Vector ADV, Nortek AS, Rud, Norway), mounted on a tripod oriented with the expected wave direction to minimize turbulence caused by the mounting structure. Longitudinal, lateral, and vertical flow velocities were measured at 64 Hz for 2 min at 5 cm depth. We first tested ε estimation from velocity spectra using inertial-subrange scaling combined with Taylor’s frozen-turbulence hypothesis.^39^ This approach was unsuccessful because the inertial subrange could not be well-resolved. We therefore estimated ε using a large-eddy scaling approach, assuming local equilibrium between turbulence production and dissipation, based on root-mean-square fluctuating velocities (after removing wave motions and high-frequency noise) and a characteristic length scale set by water depth^57^ (Supporting Text S4).

### Particle Concentrations

Particle abundance (P_A_) was measured using image-based flow cytometry with two benchtop FlowCams (Yokogawa Fluid Imaging Technologies, Inc., Scarborough, Maine), analyzed immediately after sampling. For small particles (2−20 μm area-based diameter, ABD), a FlowCam 8400 with a 100 μm flow-cell (FOV-100), a 10× objective, and a C80 syringe pump (1.00 mL) was used. Samples were run in Auto-Image mode at 0.150 mL min^−1^ and 20 fps for 15 min, capturing >1000 images; each unfixed sample was run twice and averaged for analysis. For larger particles (20−300 μm), a FlowCam CYANO (8000 series) with a 300 μm flow-cell (FOV-300), a 4× objective, and a C80 syringe pump (5.00 mL) was used at 0.900 mL min^−1^ and 6 fps for 20 min, capturing >600 images per replicate run. Data were processed using Visual Spreadsheet (VSP v4.19.3). Particle counts from both size ranges were combined, and analyses focused on particles in the 2−100 μm range.

### Statistical Analysis

To investigate spatiotemporal drivers of *k*_600_,_CH4_ *:k*_600_,_CO2_ (testing H1), and microbubble void fraction (testing H2), linear mixed effect (lme) models were fitted using the R “nlme” package.^58^ For the *k*_600_ ratio, separate models were run with microbubble void fraction (V, scaled × 10^7^), wind speed (U), turbulent kinetic energy dissipation (ε), and chemical enhancement factor (α) as fixed effects. A second model set used V as the response, with fixed effects U, ε, and P_A_. Significant predictors were combined in additional multipredictor models, with model selection based on Akaike’s Information Criterion. In all models, sampling location was included as a random intercept to account for repeated observations. Additionally, to assess nearshore areas and macrophyte stands as potential sources of microbubbles, V was modeled against water depth and transect type (reed/nonreed) using subset R1−R4 and N1−N4 with sampling day and location as random effects. Finally, linear models (lm) were applied to the M site subset to assess temporal relationships. Models were fitted by maximum likelihood, residuals were evaluated for homogeneity and normality, and outliers were assessed using Cook’s distance. Model fits were summarized using marginal *R*_m_^2^ (lme, MuMIn package^59^) and *R* ^2^ (lm). In all analyses, U was averaged over sampling hours, whereas V and ε were first averaged over replicate measurements and then over the corresponding sampling hour.

## Results

### Environmental Parameters

Surface water temperature varied from 17.6 °C at R1 to 21.1 °C at D (mean ± SD across sites: 19.1 ± 0.7 °C). The pH ranged from 8.45 at R1 to 8.79 at D (8.67 ± 0.07). Dissolved oxygen ranged from 103.2% at R1 to 120.0% at N2 (111.0 ± 3.15%), indicating net autotrophy. Hourly average wind speed varied from 0.85 m s^−1^ at M to 6.90 m s^−1^ at site D. Summary statistics of the environmental variables by sampling site, together with additional physicochemical parameters, are presented in Table S1. Mixed layer depth was ∼5 m during the sampling campaign.

### Microbubble Concentrations

Microbubble concentrations spanned 4 orders of magnitude (10^2^−10^6^ m^−3^ μm^−1^) over the 9−44 μm radius range. They decreased with radius, following power-law behavior close to the expected −3/2 scaling.^27^ Sampling sites differed in magnitude rather than in spectral slopes of bubble size distributions, with R sites exhibiting the largest magnitude, M and D sites showing midrange, and N sites showing the lowest microbubble concentrations (Figure 1a). Our measurements lie within the broader context of natural waters when compared with the literature data. The “ocean surf” zone reference bounds the upper end of expected concentrations and remains 1−2 orders of magnitude above our data. Our spectra were within or below lake reference bands and remain well above (calm) “open ocean” and “pool” baselines (references in Figure 1a).

**Figure 1:**
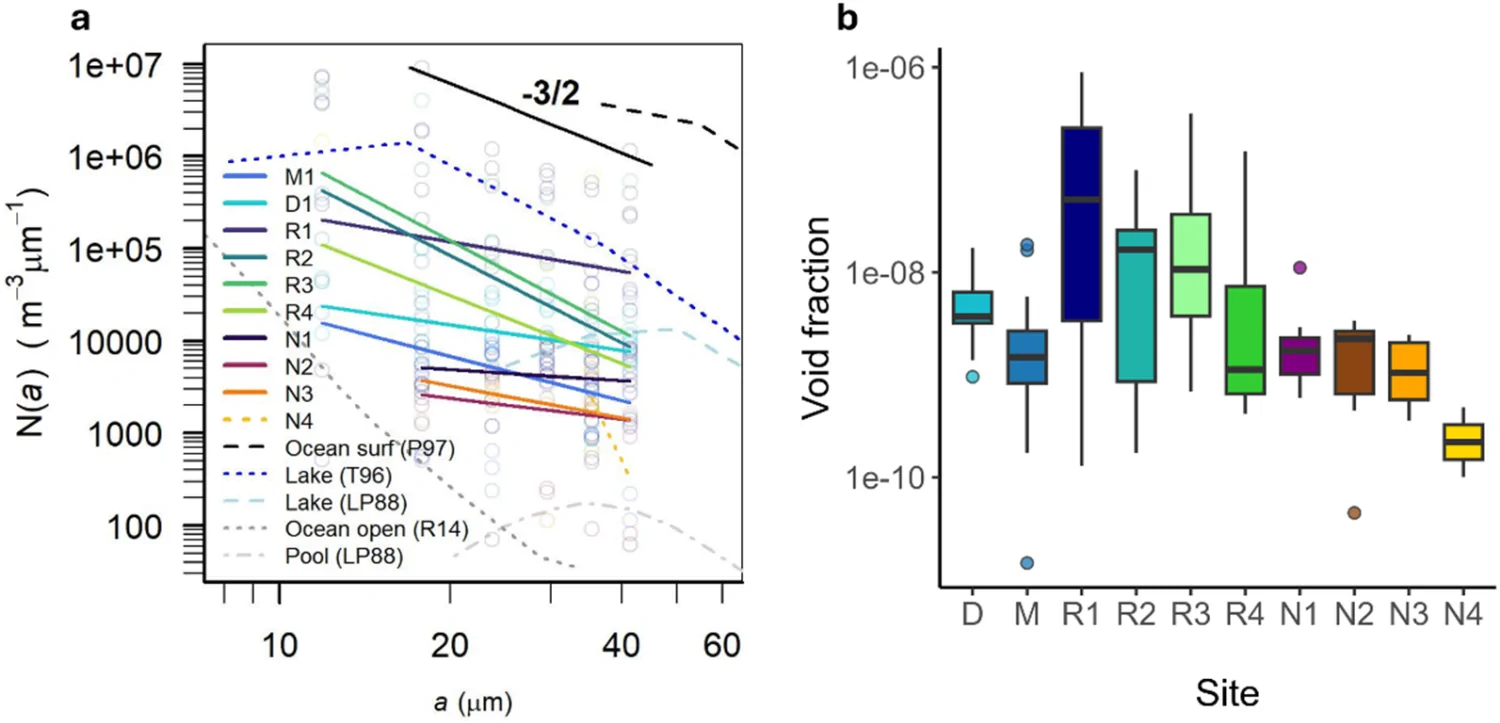
Microbubble concentration (N) vs bubble radius (a) across sites with literature comparison (a). Circles show individual observations while colored solid lines show least-squares linear regressions in log−log scale, and the colors denote sites. The dashed yellow line (N4) indicates values near the detection limit. The thick black line shows the theoretical spectral slope of −3/2, expected for wave-induced microbubble entrainment.^27^ Literature data: P97,^60^ T96,^61^ LP88,^29^ and R14.^62^ Microbubble void fraction (*a* = 9−44 μm) across sites (b). Boxes show interquartile range (IQR), whiskers 1.5 × IQR, circles outliers, and black lines the median.

Microbubble void fractions (9−44 μm radius) ranged from 1.46 × 10^−11^ at M to 8.93 × 10^−7^ at R1 (3.27 × 10^−8^ ± 1.17 × 10^−7^, Figure 1b). Intermediate levels were found at D and N sites. Void fraction decreased significantly with water depth, but the overall difference between reed and nonreed transects was not significant (Table S5). Microbubble void fraction density as a function of bubble radius is shown in Figure S5.

### Gas Concentrations and Fluxes

The near-surface water was CH_4_-supersaturated and CO_2_-undersaturated relative to the atmosphere, with concentrations ranging from 0.37 to 1.62 μM for CH_4_ (mean ± SD: 0.67 ± 0.21) and 5.30 to 14.75 μM (8.38 ± 1.89) for CO_2_, respectively (Figure S6a,b). CH_4_ and CO_2_ concentrations followed similar patterns across sites, with the highest concentrations, but also high variability, at R1 and R2, and the lowest at D and to a lesser extent at M. CH_4_ and CO_2_ concentrations at N transect sites and R3 and R4 sites were moderate.

The lake was a source of CH_4_ to the atmosphere and a sink for CO_2_ (Figure S7). The CH_4_ emissions varied from 0.16 to 4.82 mmol m^−2^ d^−1^ (1.41 ± 0.92 mmol m^−2^ d^−1^). CO_2_ uptake ranged from −0.99 to −48.94 mmol m^−2^ d^−1^ (−19.40 ± 10.50 mmol m^−2^ d^−1^). The CH_4_ and CO_2_ fluxes followed similar patterns across lake sites, increasing along the R and N transects with distance from shore and being highest at the offshore site M.

### Gas Transfer Velocities

The *k*_600_ for CH_4_ and CO_2_ ranged from 0.15 to 6.89 m d^−1^ (2.27 ± 1.33 m d^−1^) and 0.22 to 7.96 m d^−1^ (2.44 ± 1.28 m d^−1^), respectively (Figure S9). Across sites, *k*_600_ showed coherent spatial patterns for both CH_4_ and CO_2_, increasing with distance from shore along the R and N transects and being highest at the offshore M site. Individual *k*_600_,_CH4_ *:k*_600_,_CO2_ estimates varied from 0.21 to 2.93 (0.93 ± 0.39), showing no clear enhancement of *k*_600_ for CH_4_ or CO_2_, but somewhat lower ratios at littoral sites, especially R1 and R2 (Figure 2a). The *k*_600_ of CH_4_ and CO_2_ were strongly correlated (*R*_m_^2^ = 0.69), with most observations clustering near the 1:1 line (Figure 2b). The empirical fit lies well below the steeper relationship previously found in Lake Stechlin^15^ (red line), suggesting no systematic enhancement of CH_4_ transfer relative to CO_2_ under our sampling conditions.

**Figure 2:**
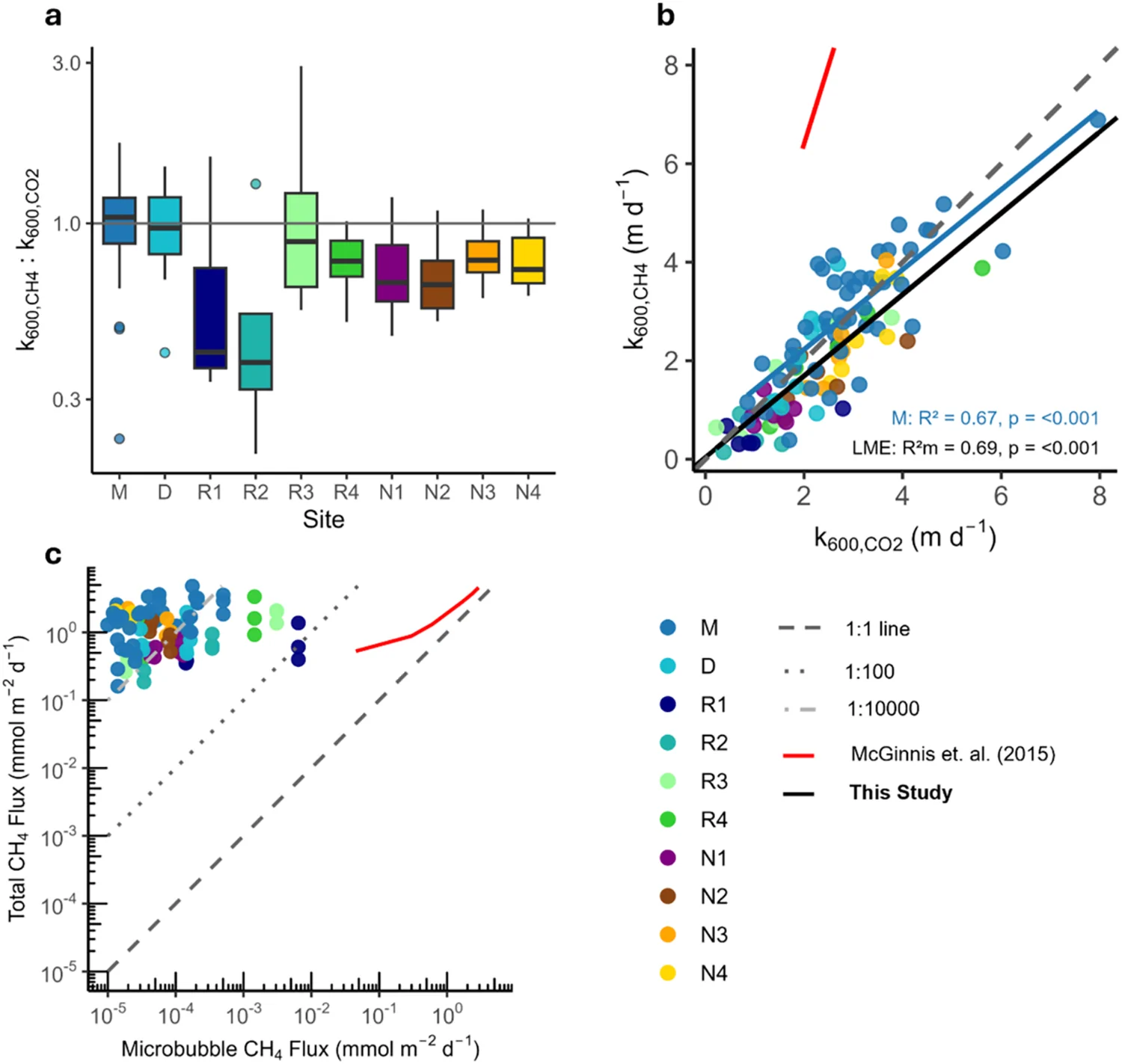
(a) *k*_600,CH4_:*k*_600,CO2_ ratios across sites; boxes: IQR, whiskers: 1.5 × IQR, center line: median, circles: outliers, and gray line: ratio = 1. (b) Relationship between *k*_600_,_CH4_ and *k*_600_,_CO2_; solid black line: linear mixed-effect fit, solid blue line: linear fit for M site (Table S2), gray dashed line: 1:1 line; and red line: published empirical relationship.^15^ (c) Microbubble-mediated CH_4_ flux vs total CH_4_ flux (log−log); gray dashed: 1:1 line, dotted line: 1:100; dot-dashed line: 1:10000; and red line: theoretical relationship from McGinnis et al.^15^ Colored boxes/points denote sites across all panels.

The theoretical maximum microbubble-derived CH_4_ fluxes were two to 5 orders of magnitude lower than total CH_4_ fluxes (Figure 2c). Microbubble CH_4_ fluxes spanned from 9.9 × 10^−6^ to 6.4 × 10^−3^ mmol m^−2^ d^−1^, whereas total CH_4_ fluxes ranged from 0.16 to 4.82 mmol m^−2^ d^−1^, implying that microbubble contributions to total fluxes ranged from 0.0005% at M to 1.61% at R1 (0.05 ± 0.19%). While previous theoretical estimates suggested microbubbles contributed approximately 8−63% to total CH_4_ fluxes in Lake Stechlin^15^ (Figure 2c), microbubbles were a negligible CH_4_ flux source under our sampling conditions.

### Drivers of *k*_600_ Ratios

Averaged over each sampling occasion, apparent *k*_600_ ratios ranged from 0.28 to 1.57, with no clear relationship with microbubble void fractions (Figure 3a). Similarly, they showed no systematic relationship with ε, indicating a limited direct effect of turbulence across all sites (Figure 3b). Average wind speed was the strongest predictor of *k*_600_ ratios, with significant positive relationships across sites (*R*_m_^2^ = 0.30, *p* < 0.001) and at site M (*R* ^2^ = 0.33, *p* < 0.05, Figure 3c). The wind-speed effect was weaker than previous observations from Lake Stechlin.^15^ A weak but significant negative relationship was observed for *k*_600_ ratios and chemical enhancement factor (α) across sites (*R*_m_^2^ = 0.12, *p* < 0.03), but not at site M (Figure 3d), with α ranging from 1.04 to 9.56. Overall, the wind-speed effect on *k*_600_ ratios resulted largely from both wind-induced turbulence and suppression of chemical enhancement, as indicated by significant correlations of ε and α vs wind speed (Figure S11), and insignificance of α effects when added to the wind speed model (Table S3a).

**Figure 3:**
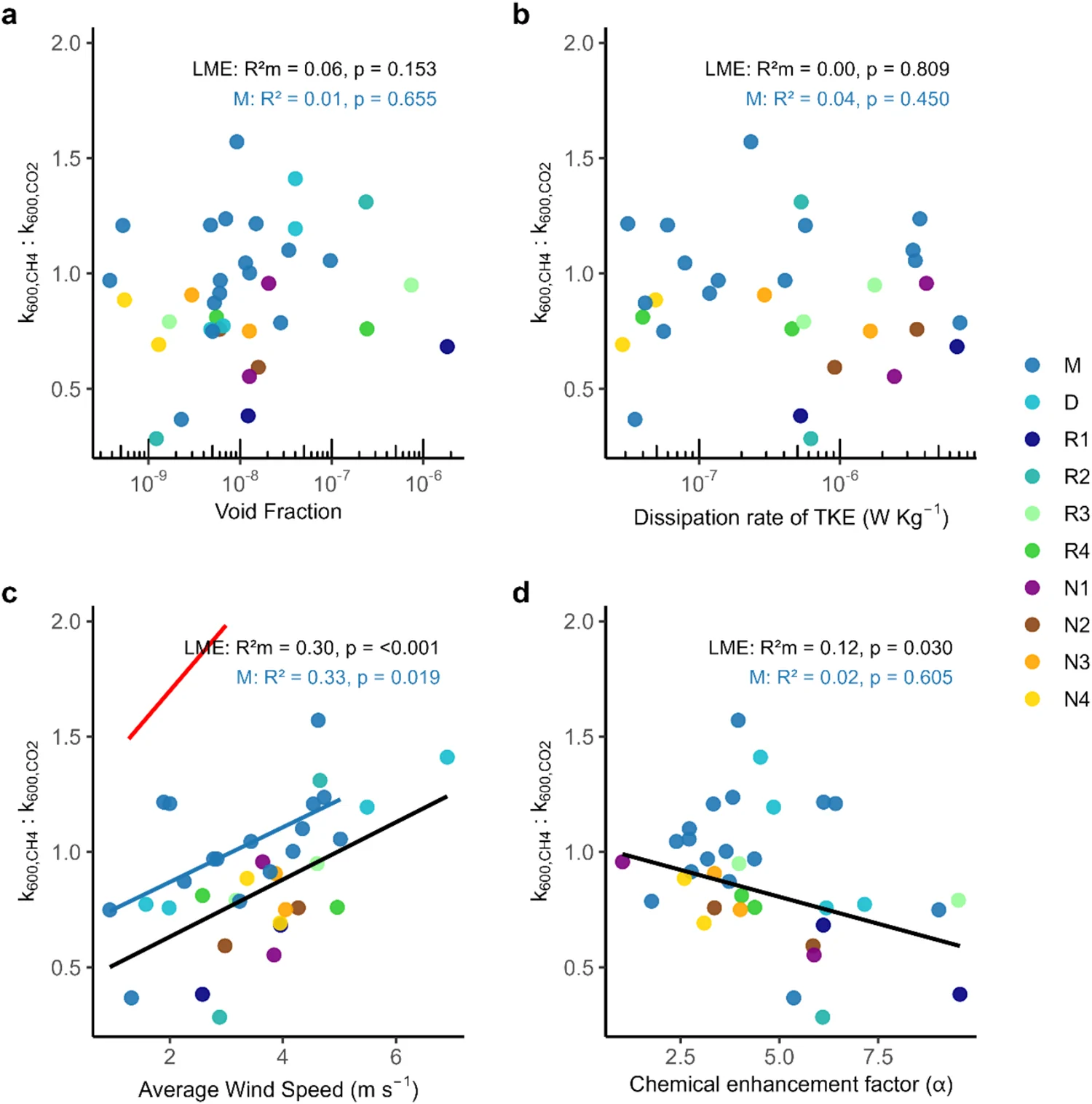
Relationships between *k*_600_ ratios and (a) microbubble void fraction, (b) TKE dissipation rate, (c) average wind speed, and (d) chemical enhancement factor (α) across sites. In (c), the red line shows a published empirical relationship from Lake Stechlin.^15^ Solid black and blue lines show linear mixed-effects models (LME, all sites) and linear models (M site), respectively, with significant effects (*p* < 0.05). Model fits and significance are given by marginal *R* ^2^ (*R*_m_^2^) and *R* ^2^, respectively, and *p*-values (Table S3a,b), following the line’s color code.

### Drivers of Microbubble Void Fraction

Microbubble void fraction increased weakly, but significantly with average wind speed, although the relationship was relatively weak (Figure 4a; *R*_m_^2^ = 0.18, *p* = 0.008) and ε (Figure 4b; *R*_m_^2^ = 0.24, *p* = 0.004). They also showed a positive relationship with particle abundance (Figure 4c; *R* ^2^ = 0.18, *p* = 0.03), which, however, became marginally insignificant (*p* = 0.076) when added to the wind speed model (Table S4a), was sensitive to influential observations (R1 and R3, Cook’s distance = 0.62 and 0.40) and was no longer significant after their removal (not shown). At the M site, any of the predictors had generally weaker effects.

**Figure 4:**
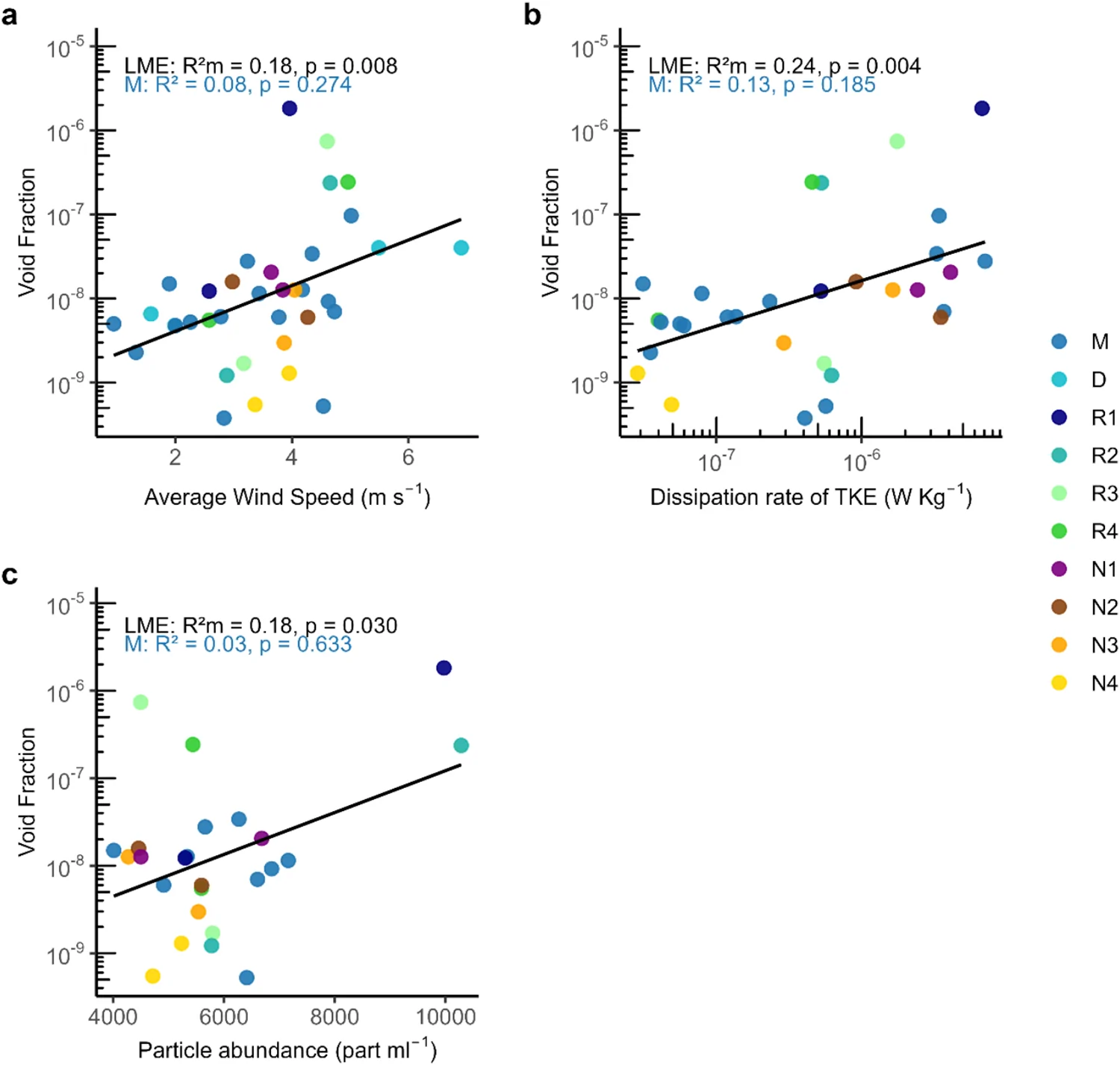
Relationships between void fraction and (a) average wind speed, (b) dissipation rate of TKE, and (c) particle abundance (2−100 μm). Solid black lines show linear mixed-effects models (LME) across sites with significant effects (*p* < 0.05). The fits and significance of LME and linear models (M site only) are given by marginal *R* ^2^ (*R*_m_^2^) and *R* ^2^, respectively, and *p*-values (Table S4a,b).

## Discussion

### Testing the Microbubble Hypothesis

The microbubble hypothesis suggests elevated *k*_600_,_CH4_ relative to *k*_600_,_CO2_ because microbubbles preferentially carry more sparingly soluble gases (e.g., more CH_4_ than CO_2_) through the surface boundary layer and vent at the air−water interface, partially bypassing water-side diffusive resistance. Consequently, *k*_600_,_CH4_ should exceed *k*_600_,_CO2_ when microbubble fluxes are sufficiently high and other factors that differentially affect apparent gas transfer are negligible. This mechanism has been invoked to explain enhanced apparent *k*_600_ of CH_4_ relative to CO_2_ in Lake Stechlin^15^ and elsewhere.^17^

Across sampled sites and sampling days, we detected microbubbles in the 9−44 μm range over 4 orders of magnitude in void fraction. Yet, *k*_600,CH4_ *:k*_600,CO2_ was consistently close to 1 (H1a, Figure 2b), there was no positive correlation between *k*_600,CH4_ *:k*_600,CO2_ and microbubble void fraction (H1b, Figure 3a), and microbubbles contributed theoretically only ∼0.05% to total CH_4_ fluxes (H1c, Figure 2c). Collectively, these results suggest that microbubbles, despite measurable concentrations, did not substantially enhance CH_4_ relative to CO_2_ transfer under the conditions prevailing at Lake Stechlin during our measurements, generally rejecting hypothesis H1.

It is noteworthy that our results contradict previous findings from Lake Stechlin,^15^ that reported consistent *k*_600,CH4_ *:k*_600,CO2_ > 1 (Figure 3b) from which they inferred strong microbubble-induced enhancement of CH_4_ over CO_2_ exchange, with microbubbles theoretically contributing 8 to 63% to the total CH_4_ flux. While the contrasting result between the two studies may partly reflect differences in the methods used for microbubble CH_4_ flux estimation, they likely also reflect contrasting wind conditions and lake-state regimes. The previous observations^15^ were dominated by easterly winds of 4−6 m s^−1^, conducive to more intense bubble entrainment^63^ and likely to lower chemical enhancement of CO_2_ through stronger turbulence. In contrast, wind speeds in our study were generally lower, clustering between 2−4 m s^−1^, and varied in direction (Figure S13). These conditions were less favorable for bubble entrainment but more conducive to chemical enhancement of CO_2_ transfer. Under these conditions, the negligible contribution of microbubbles limited the enhancement of *k*_600,CH4_, whereas chemical enhancement of CO_2_ increased the apparent *k*_600,CO2_. Together, these effects, along with other processes not explicitly considered here, likely shifted the *k*_600,CH4_ *:k*_600,CO2_ ratio toward unity or below unity in most of our samples. In addition, the previous study was performed at the beginning, while our study was performed after a phase of rapid eutrophication at Lake Stechlin, with elevated phosphorus and algal biomass, reduced water clarity, and warmer surface waters.^64^ The changed lake conditions could have altered near-surface microstratification,^65,66^ affecting bubble size distributions, penetration depth,^67^ and microbubble transport toward the surface.^68^ They may have also changed surfactant accumulation and composition, which can affect CH_4_ uptake into bubbles^28^ and microbubble lifetimes in the surface boundary layer,^69^ potentially altering bubble-mediated CH_4_ exchange with the atmosphere. We indeed observed bubble accumulation at the surface in the reed transect, coinciding with elevated microbubble concentrations (Figures 1b, S3), but it remains unclear whether microbubble exchange was hampered more in the surface boundary layer under our sampling conditions, relative to the conditions experienced previously.^15^

These considerations help reconcile our findings with the previous study.^15^ If microbubbles were indeed the main driver of previously observed high *k*_600_ ratios,^15^ then microbubble concentrations, and the associated CH_4_ efflux, must have been substantially higher than in our case. This appears plausible in principle, as previously published studies from Lake Biwa and ocean surf zones report microbubble void fractions that are up to 2 orders of magnitude higher than the highest values observed in our study (Figure 1a). However, these cases were associated with substantially stronger forcing, with wind speeds up to ∼12 m s^−1^ in Lake Biwa^61^ and ∼12−15 m s^−1^ in the ocean surf-zone study.^60^ In our data set, microbubbles accounted for ∼0.05% of the total CH_4_ flux, implying modest air fluxes through microbubbles. A simple scaling suggests that a 10−100-fold increase in microbubble void fraction and emission would translate into a contribution of ∼0.5−5% of total CH_4_ flux. Thus, our results do not contradict the microbubble hypothesis in general, rather, they indicate that under the relatively low-wind, posteutrophication conditions encountered during our campaign, microbubbles contributed only marginally to CH_4_ transfer, and they further suggest that the large microbubble-driven fluxes inferred previously^15^ are difficult to reconcile with wind speeds of 4−6 m s^−1^ unless additional processes, such as enhanced production of bubble-stabilizing surfactants or elevated biological gas production, substantially increased microbubble production and/or persistence. In addition, other processes that differentially affect apparent *k*_600_ ratios may also have contributed to the elevated *k*_600_ ratios previously attributed to microbubble-mediated CH_4_ transfer.

### Alternative Explanations for Intergas Differences in *k*_600_ Ratio

Across aquatic systems, *k*_600_ values are not always directly comparable among gases, with reported *k*_600_ ratios spanning above and below unity, depending on system type, turbulence regime, and the operational definition of near-surface gas concentrations needed to calculate *k*_600_.^17^ Importantly, gas-specific near-surface biogeochemistry and sampling-depth biases can affect the apparent *k*_600_ derived from paired flux and near-surface concentration measurements.^17^ In our study, despite low microbubble influence, we found the *k*_600_ ratio to increase with wind speed across all sites and TKE dissipation at the M site (Figure 3b,c). This observation calls for alternative explanations for enhanced CH_4_ exchange under more turbulent conditions. We propose three such alternative mechanisms that may co-occur in Lake Stechlin and summarize those in Figure 5.

**Figure 5:**
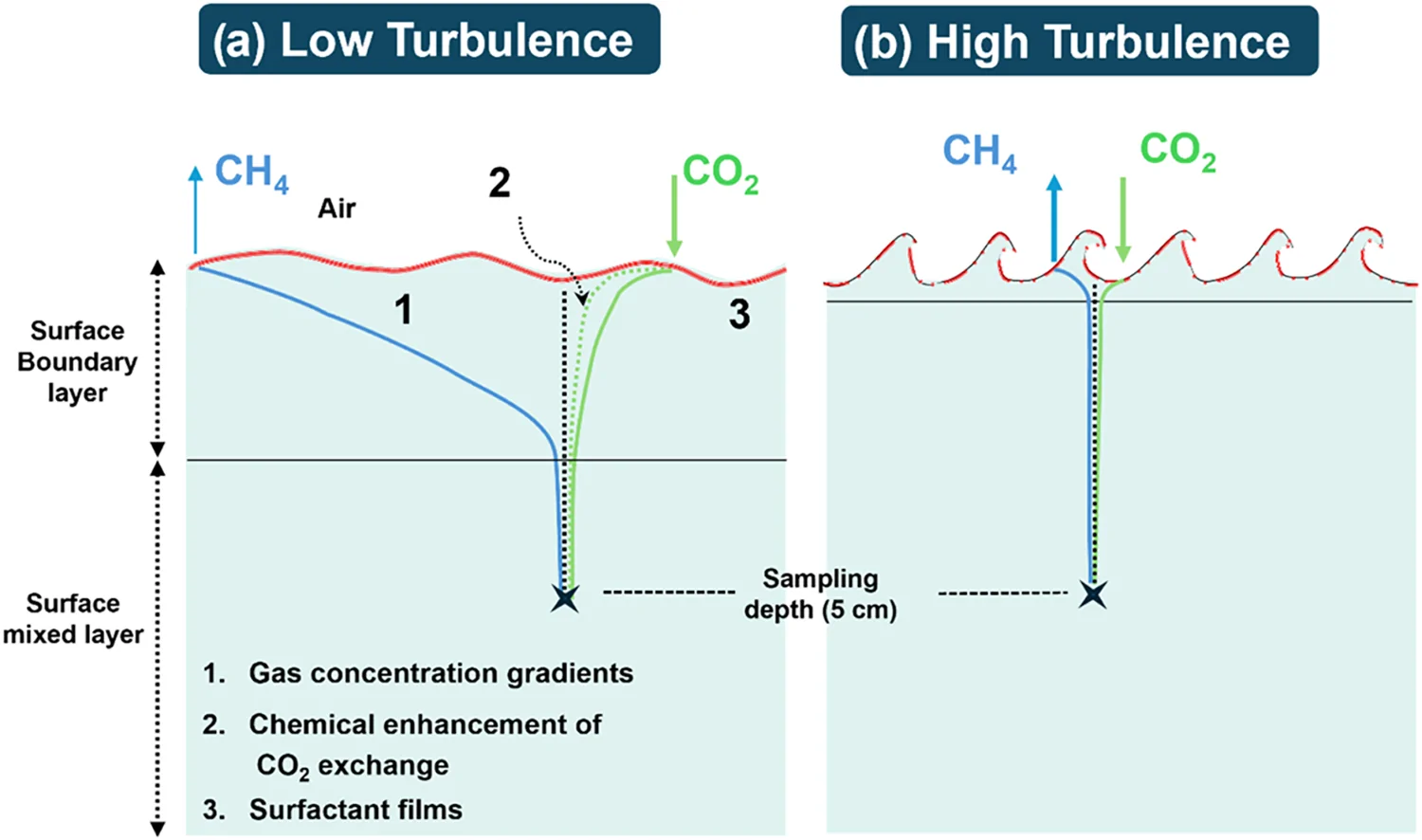
Conceptual diagram of the turbulence-driven decoupling of *k*_600_,_CH4_ and *k*_600_,_CO2_ across the air−water interface via: (1) near-surface gas concentration gradients, (2) chemical enhancement of CO_2_ exchange, and (3) surfactant films. (a) Low turbulence: Thick surface boundary layer and CH_4_ degassing create a steep gradient between the interface and sampling depth, while CO_2_ (buffered by carbonate equilibria and higher solubility) maintains a smaller gradient despite net influx. A thicker, more coherent surfactant layer (red line) may favor CH_4_ exchange over CO_2_. (b) High turbulence: Wind and wave-driven mixing thins the boundary layer, reducing gradients, disrupting or dispersing surfactant films, and enhancing transfer of both gases. Arrows indicate flux direction, blue/green lines show CH_4_/CO_2_ gradients, and black dotted lines show uniform reference profiles.

#### Mechanism 1: Sampling Bias in Near-Surface Concentration Gradients

CH_4_ is less soluble and relatively inert compared to CO_2_, which participates in the carbonate system. For a given air−water flux, this means that depletion of CH_4_ in the surface boundary layer can be stronger than for CO_2_. Under low-turbulence conditions, weak vertical mixing allows the surface boundary layer to become partially decoupled from the water at sampling depth (here ∼5 cm)^70^ (Figure 5a). A rapid degassing of CH_4_ from this thin layer may then create a steep vertical gradient between the interface and sampling depth, whereas CO_2_, buffered by carbonate equilibrium reactions and higher solubility, may maintain a comparatively smaller gradient despite its net influx. When “apparent” *k*_600_ is calculated from fluxes and the concentration difference between air and 5 cm depth, such unresolved near-surface gradients lead to an over- or underestimation of the true driving gradient. Because this bias is expected to be larger for CH_4_ than for CO_2_, CH_4_ measurements at 5 cm depth will overestimate surface conditions, and *k*_600_,_*CH4*_ will be underestimated more strongly at low turbulence, yielding *a k*_600_ ratio <1. As turbulence increases, enhanced mixing thins the surface boundary layer and couples it more tightly to the underlying water, reducing the gas-specific gradient bias and allowing the *k*_600_ ratio to increase toward values >1 (Figure 5b).

#### Mechanism 2: Chemical Enhancement of CO_2_ Exchange

Chemical reactions of dissolved CO_2_ with the carbonate system within the surface boundary layer can enhance the effective transfer velocity of CO_2_ relative to CH_4_. This chemical enhancement, commonly expressed via an enhancement factor α, increases with pH and decreases with turbulence as the residence time in the boundary layer shortens.^71,72^ CH_4_ is chemically inert on the time scales of air−water exchange (α ≈ 1), so chemical enhancement systematically increases *k*_600_,_CO2_ relative to *k*_600_,_CH4_, and therefore tends to depress *k*_600,CH4_ *:k*_600,CO2_. This mechanism has been suggested as one contributor to higher apparent *k*_600,CH4_ *:k*_600,CO2_ in some lakes and rivers.^17^ In our study, *k*_600,CH4_ *:k*_600,CO2_ decreased significantly with α across sites (Figure 3d). Chemical enhancement thus lowers *k*_600,CH4_ *:k*_600,CO2_ at low wind speeds and turbulence, but is less pronounced at higher levels (Figure 5).

#### Mechanism 3: Suppressed Gas Exchange through Surfactant Films

Natural surfactants that accumulate in the surface microlayer can suppress molecular diffusion and dampen small-scale capillary−gravity waves and near-surface turbulence, thereby reducing gas transfer velocities by up to 20−70% at low to moderate wind speeds,^11,73–75^ depending on surfactant type. Laboratory and field experiments show that this suppression is strongest at low wind, when surfactant films remain more coherent, and becomes weaker with increasing wind stress and wave breaking, which tends to disrupt or disperse the films.^76,77^ Importantly, because surfactants primarily suppress gas exchange by increasing water-side resistance, their effect is expected to be greater for gases with lower solubility than for those with higher solubility.^78^ By analogy, we cautiously propose that surfactant-induced suppression of *k* under low-turbulence conditions could be stronger for CH_4_ than for CO_2_ (Figure 5a). However, because surfactant properties were not measured in this study, this mechanism remains plausible but untested and requires further mechanistic validation. A surfactant-stabilized, low-turbulence surface would then preferentially suppress *k*_600_,_CH4_, lowering the *k*_600_ ratio at low wind speeds. With increasing turbulence, surfactant films may be partially broken up, and the suppression becomes less gas specific, contributing to the observed increase in *k*_600_ ratio with increasing wind speed (Figure 5b).

### The Controls on Microbubble Occurrence in Lake Stechlin

#### Wind Speed and Turbulence

In Lake Stechlin, we found measurable yet low microbubble void fractions. The weak but significant increase of void fractions with wind speed and ε (Figure 4a,b), together with the spectral slopes of microbubble concentrations being close to −3/2 (Figure 1a), suggest microbubbles to be mainly produced by turbulence-driven air entrainment through breaking or steepening of wind-forced surface waves. Laboratory and numerical work on breaking waves shows that the volume of entrained air and the bubble size distribution scale with ε: stronger free-surface turbulence entrains more bubbles and drives them deeper in the water column.^79–81^ In our study, the highest void fractions of >10^−8^ occurred at elevated ε (>10^−7^ W kg^−1^), consistent with episodes of intense near-surface turbulence from wind-waves and shear. Conversely, under relatively calm conditions with low ε (∼10^−8^ W kg^−1^), we still observed void fractions around 5 × 10^−9^ that likely reflect bubbles left over from earlier wave events that can persist for seconds to hours through stabilizing surfactants^82,83^ (Figure 6a). This hysteresis between bubble presence and turbulence could explain part of the substantial scatter in the relationships of void fraction with wind speed and turbulence (Figure 4a,b), consistent with marine observations at similarly moderate winds.^26,84,85^ The scatter could also be explained by elevated loss of bubbles through surface bursting under more turbulent conditions, or the presence of alternative microbubble sources that are less affected by turbulence (Figure 6b).

**Figure 6:**
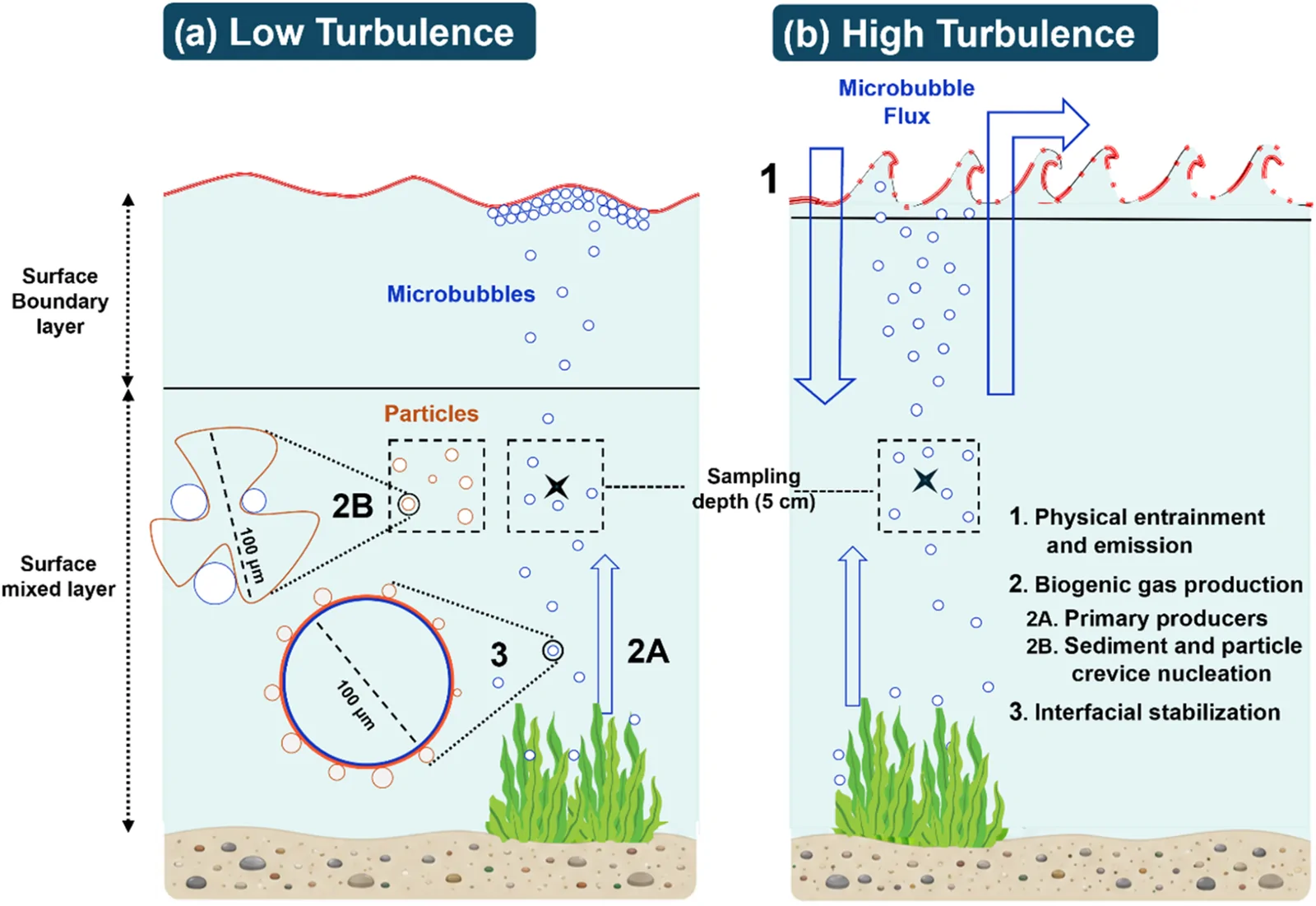
Conceptual diagram of controls on microbubble occurrence: (1) turbulence-driven physical entrainment/emission, (2) biogenic production (2A; primary producers and 2B; sediment and particle crevice nucleation), and (3) interfacial stabilization by surfactants and particles. (a) Low turbulence: A thick surface boundary layer and coherent surfactant layer (red line) may favor microbubbles (blue circles) retention below the surface, limiting surface exchange. (b) High turbulence: Wind and wave-induced mixing thins the surface boundary layer, disrupts or disperses surfactant films, and promotes entrainment from the air−water interface and scavenging from primary producers/sediments, increasing microbubble abundance at sampling depth, and enhancing surface transfer and gas exchange. Arrows indicate microbubble pathways (scaled by flux); dashed box highlights microbubble population at sampling depth.

#### Nearshore Areas

We found elevated microbubble void fractions toward shallower depths near the lake shore and higher variability, but no difference between reed and nonreed areas (Figure 1b). This pattern suggests that shallow littoral areas may act as local sources or modify the processes that govern microbubble formation and loss (although we cannot fully exclude that part of the enhancement reflects bubbles released by plant or sediment disturbance during sampling). Shallow littoral zones can influence bubble occurrence in different ways. *First*, gas bubbles can form on submerged plants and epiphytic algae during photosynthesis in oxygen supersaturated water and be released periodically into the water column.^34,35^ Even though these bubbles are initially dominated by O_2_, they also strip other dissolved gases.^28^ In our study, lake water was O_2_ oversaturated (103−120%), consistent with intense photosynthesis, relative to respiration, and potential for bubble formation. *Second*, shallow littoral zones with macrophytes tend to attenuate waves and dampen local white capping, which could reduce microbubble entrainment, whereas canopy edges may enhance shear and turbulence and thus promote local entrainment and breakup of bubbles.^86^ *Third*, shallow sediments are hotspots of CH_4_ cycling, especially when covered with macrophytes, which alter CH_4_ ebullition pathways through various biogeochemical and hydrodynamic processes.^87^ Such changes in ebullition may in turn affect microbubble populations, for example, if rising macrobubbles scavenge pre-existing microbubbles, or if their fragmentation or surface rupture produces smaller, longer-lived “daughter” bubbles.^88^ Whether these mechanisms result in a net release or reduction of microbubbles in the littoral zone, and how their relative importance depends on turbulence and other local conditions in Lake Stechlin, remains uncertain. Our field observations indicate elevated microbubble void fractions in shallow areas, but do not allow us to distinguish among these competing processes or quantify their relative contributions. Collectively, these mechanisms therefore illustrate plausible pathways by which shallow littoral zones, particularly those with dense macrophyte stands, could influence microbubble cycling (Figure 6).

#### Particle-Mediated Microbubble Formation and Stabilization

The weak yet positive correlation of microbubble void fractions with particle abundance (Figure 4c) may suggest the production and/or stabilization of microbubbles through microscale particles in Lake Stechlin^89^ (Figure 6a). Smaller particles or surfactants suspended in the water can be adsorbed to the microbubble surface, forming a stabilizing interfacial film that prevents bubble dissolution or coalescence, which can prolong microbubble lifetime.^83^ Small inhomogeneities in larger particles may also serve as microbubble formation sites through crevice-type nucleation if local dissolved gas concentration exceeds a critical supersaturation threshold to overcome the surface tension energy barrier.^90^ The crevice model of bubble nucleation yields the supersaturation ratio $S_{crit}=\frac{C_{c}}{C_{w}}=1+\frac{2\sigma }{R\rho }$ , where *C*_c_ is the gas concentration in the crevice, *R* is the radius of the curvature of the crevice, and σ ∼0.072 N/m is the surface tension. For *R* = 1 μM and *C*_w_ = 0.7 μM, *C*_local_ would be ∼1 mM, or more than 1000 times enrichment compared to bulk water. While bubble nucleation is unlikely in bulk water, microenvironments such as sediment crevices,^91^ suspended particles,^92^ or biofilm interstices may locally accumulate enough CH_4_ to nucleate bubbles.^93^ These mechanisms remain to be confirmed in future studies.

Accurate CH_4_ and CO_2_ flux estimates from lakes require mechanistic insight into the divergence of *k*_600_ between CH_4_ and CO_2_ under field conditions and the role of microbubbles in enhancing the CH_4_ *k*_600_. Our results indicate that, in Lake Stechlin under low-wind, posteutrophication conditions, microbubbles were not a first-order control on the difference between *k*_600,CH4_ and *k*_600,CO2_, and contributed insignificantly to total CH_4_ fluxes. Moreover, *k*_600,CH4_ remained close to, or slightly below, *k*_600_,_CO2_. This aligns with growing evidence that Schmidt-number scaling alone cannot reliably be used to convert *k* among gases: some systems show elevated apparent CH_4_ transfer consistent with hypothesized microbubble-mediated exchange playing a dominant role,^15^ whereas others show equal or even higher *k*_600,CO2_, indicating that other processes can dominate under different conditions.^17^ Our results further highlight the potential influence of sampling bias in near-surface gas concentration, chemical enhancement of CO_2_ exchange, and surfactant-mediated suppression of gas transfer in shaping apparent *k*_600_ ratios. We also identify plausible controls on microbubble occurrence, including turbulence, biogenic production, and interfacial stabilization by surfactants and particles. Our process-based comparisons suggest that microbubble-mediated gas exchange may become important under more energetic or site-specific conditions, indicating that the microbubble hypothesis may still apply in other systems and in Lake Stechlin under higher levels of turbulence. Future studies should adopt a process-based framework that integrates *k* estimates with concurrent observations of microbubble abundance, near-surface gas concentration gradients, and surfactant compositions. Expanding such coordinated measurements across broader turbulence regimes will be critical to disentangling possible mechanisms that may affect *k*_600_ differently for different gases.

## Supplementary Material





## Notes

Source data contain data needed to reproduce results in the manuscript and supporting (XLSX)
Acoustic microbubble measurements (Text S1), dissolved CH_4_ and CO_2_ measurements (Text S2), and the calculation of turbulent kinetic energy dissipation rates (ε) from Acoustic Doppler Current Profiler (ADCP) data (Text S3), Acoustic Doppler Velocimetry (ADV) data (Text S4), and bubble gas exchange modeling (Text S5). The figures include a map of the Lake Stechlin and sampling locations (Figure S1), photographs of the littoral transects (Figure S2), the ABS hydrophone setup and evidence of bubble stabilization by surfactants (Figure S3), the floating and flying chamber deployments (Figure S4), microbubble void fraction density per unit radius (Figure S5), site-wise distributions and intercomparisons of CH_4_ and CO_2_ concentrations (GC vs analyzer measurements including Picarro HS; Figure S6), flux distributions and cross-chamber comparisons (Figures S7 and S8), gas transfer velocity (*k*_600_) distributions and comparison of *k*_600,CH4_ and c derived from floating and flying chamber fluxes (Figures S9−S10), relationship between wind speed, dissipation rate of TKE, chemical enhancement factor and particle abundance (Figure S11), selected examples of ADV velocity−wavenumber spectra illustrating the spectral fitting approach and noise-floor breakpoints (Figure S12), average wind speed and wind direction (Figure S13), and modeled microbubble equilibration and residence times (Figure S14). The tables include a summary of environmental, physical, and aqueous chemical parameters (Table S1) and summaries of mixed effect models (Tables S2a, S3a, S4a, and S5) and linear models (Tables S2b, S3b, and S4b) (PDF)
