# Supplementary material for: Evaluating the Microbubble Hypothesis: Apparent Air−Water CH4 and CO2 Gas Transfer Velocities in Relation to Microbubble Concentrations in a Meso-Oligotrophic Lake
Source: Environ Sci Technol. 2026 Sep 7;60(37):26476–89. doi: 10.1021/acs.est.6c05689 (PMC13614181; doi:10.1021/acs.est.6c05689)
Supplement: Supplementary file 1 [file es6c05689_si_002.pdf]

Supporting Information

Evaluating the Microbubble Hypothesis: Apparent Air–Water CH_4 and CO_2 Gas Transfer Velocities in relation to microbubble concentrations in a Meso-Oligotrophic Lake

Mohammad Atif Khan[1*], Philipp S. Keller[2], Georgiy Kirillin[4], Hans-Peter Grossart[2,3], Stella A. Berger[2], Martin Lienenlüke[2], Jens C. Nejstgaard[2], Mats Öquist[1], Marcus Klaus[1†]

[1]*Department of Forest Ecology and Management, Swedish University of Agricultural Sciences, Skogsmarksgränd 17, 90183 Umeå, Sweden*

[2]*Department of Plankton and Microbial Ecology, Leibniz Institute of Freshwater Ecology and Inland Fisheries (IGB), 16775 Stechlin, Germany*

[3]*Institute of Biochemistry and Biology, Potsdam University, 14469 Potsdam, Germany*

[4]*Department of Ecohydrology and Biogeochemistry, Leibniz Institute of Freshwater Ecology and Inland Fisheries (IGB), 12587 Berlin, Germany*

Corresponding authors: ∗atif.khan@slu.se, +46 73 997 5817,

†marcus.klaus@slu.se, +46 70 589 3333

Content of Supporting Information

Text S1 – S5

Figure S1 – S14

Table S1 – S4

Supplementary material and methods

Text S1 Microbubble measurements: Acoustic Bubble Spectrometry

We measured microbubble number and volume (void fraction) size distributions using a Weather Resistant Acoustic Bubble Spectrometer®© (ABS) (Dynaflow Inc., Jessup, MD). The ABS utilizes the effect of bubbles on the propagation of acoustic waves. The system consists of sets of hydrophones which transmit and receive short single-frequency bursts of acoustic waves at different frequencies in a bubbly sample medium. The received signals are analyzed against those from a reference medium to determine frequency dependent sound speed ratios and attenuations. The bubble size distribution is deduced from this data by solving two Fredholm Integral Equations of the first kind[1].

Our ABS system is based on laboratory versions of the ABS[1–3], but was redesigned here for field applications. It consists of two sets of hydrophones with surface areas of 5x5 cm^2 and resonance frequencies of 50 KHz and 250 KHz. Unfortunately, the 50 KHz set malfunctioned during our sampling and hence only data from the 250 KHz set is shown here. The hydrophones are inserted in streamlined PVC holders, which in turn are connected with an adjustable handle, allowing variable distance between transmitter and receiver hydrophones. Here, we used a distance of 7.5 cm. The signals are generated and received with a PC based high-speed data acquisition (DAQ) card (NI PXIe-1071 PXI Chassis with NI PXIe-8301 Remote Control Module and NI PXI-6115 DAQ Multifunction I/O Module, National Instruments, Texas, USA). The emitted signals are routed to the desired hydrophone pairs using a multiplexer switch and the received signals are boosted by a signal amplifier. In order to optimize signal strength and resolution, we used the iterative auto-calibration function available in the ABS system which adjusts the signal output from the DAQ card for the frequency-dependent amplitude response of the hydrophones, and the DAQ measurement range for the signal amplitude range[4].

The accuracy of the ABS has been validated with optical methods in laboratory settings for void fractions up to $3x10^{-3}$ [4]. We regard the system to also be accurate under field conditions under the following assumptions: (1) the reference medium we use to normalize sound speed and attenuation is representative for the sample medium; (2) particles are not as sensitive to acoustic signals as gas bubbles. To meet assumption (1), we used lake water as a reference medium that was sampled in a 5 L bucket before and after sampling at each sampling location, after degassing bucket water for 10 min. This time was assumed to be enough to make even the smallest detectible bubbles to rise to the surface, given their rise velocity of ~0.3 mm s^{-1} [5]. We kept the bucket with reference water in contact with the lake water to assure similar temperature. Assumption (2) is likely fulfilled for small-sized particles, including algae and microorganisms, because the acoustic cross-section of a gas bubble is at least an order of magnitude larger than that of particles of the same size due to the compressibility and resonance characteristics of gas bubbles in a liquid[6]. However, if the sample volume contains large objects (e.g. leaves, macrophytes), the measurements may be significantly compromised as they can introduce considerable attenuation unless the reference signals were obtained under the same conditions. To avoid false positive bubble detections that would occur in this case, we visually monitored the sampling volume during measurements for intrusion of such objects.

During each sampling occasion, we performed and averaged over 10 subsequent data acquisition cycles with 44 emission pulses from 70 to 500 KHz and a bin size of 10 KHz. This setup allowed us to target bubble radii of about 9-44 µm with a bin size of 5.9 µm. After data acquisition, we carefully screened all sound signals and would reject acquisitions with signals that had a signal-to-noise ratios < 1.5, or

sound speed ratios <0.1 or >5. None of our measurements had to be dropped for these reasons. As the detection limit of the ABS depends on bubble size and the environmental conditions (Chao-Tsung Hsiao, Dynaflow Inc.®, personal communication), we evaluated it empirically and found bubble detection starting to be compromised for counts below ~1000 m^{-3} μm^{-1}, which corresponds to void fractions of ~10^{-10} m^3 m^{-3} μm^{-1}.

Text S2 Dissolved CH_4 and CO_2 measurement

To quantify the dissolved CH_4 and CO_2 concentrations in the near-surface water, and to internally assess method-specific biases, we employed three distinct approaches. For the *first* method, offsite gas chromatography, 5 mL of water free of visible bubbles was collected at a depth of 5 cm and immediately injected via syringe into a 22 mL glass vial prefilled with N_2 at atmospheric pressure and sealed with a rubber septum. The vials contained 0.1 mL of 85% H_3PO_4 to lower pH below 2 and shift the carbonate equilibrium toward CO_2. Samples were maintained at room temperature and in darkness during transport to the laboratory where they were stored at 4°C and in darkness until analysis within 1–2 weeks after sampling. Headspace CO_2 (as dissolved inorganic carbon) and CH_4 concentrations were analysed using a Gas Chromatograph equipped with a Flame Ionization Detector (Clarus 580, PerkinElmer, Shelton, CT, USA), a methanizer operating at 375°C, and an autosampler (PerkinElmer TurboMatrix 110 headspace sampler). Headspace partial pressures of CH_4 and CO_2 were converted to dissolved gas concentrations using temperature-dependent equations[7–9]. CO_2 concentrations in the vials were converted to field conditions using pH and temperature using carbonate equilibrium equations[10]. For the *second* method, onsite continuous measurements, dissolved CO_2 and CH_4 were measured continuously using a gas equilibrator (FaRAGE)[11] coupled with a portable greenhouse gas analyser (GasScouter 4301, Picarro, USA). Dissolved CO_2 and CH_4 was measured by lowering a hose into the water and held for 1 min. Lake water was pumped to the FaRAGE continuously and measured at 1 Hz frequency to allow at least 40 data points to be collected and averaged. For the *third* method, onsite headspace measurements, lake water was filled into a 140 mL plastic syringe, and a 40 mL headspace was created with helium gas. After 2 min of rigorous shaking, the headspace sample gas was injected to pre-evacuated bags for storage. The gas samples were analysed in the lab using a greenhouse gas analyser (GasScouter 4301, Picarro, USA). While the offsite gas chromatography method was strictly synchronized with the flux chamber sampling, the other methods were in most cases performed within a one-hour interval of the first method at each site.

Supplementary Fig. S6b compares off-site GC measurement with on-site FARAGE and Picarro HS for CH_4 and CO_2. CH_4 agrees well across most sites, with larger deviations at some R-sites (notably FARAGE measurement at R3), while CO_2 is generally within ~±2 μM of GC measurement, except at R1 where both on-site methods diverge, likely due to handling effects under strong heterogeneity or method bias. Overall, the field methods show no consistent offset and broadly match GC measurements (given their imperfect temporal matching); therefore, all results reported in the remainder of the paper are based on offsite GC measurements.

Text S3 Calculation of turbulent kinetic energy dissipation from Acoustic Doppler Current Profiler (ADCP)

At the mid-lake site M, dissipation rates of turbulent kinetic energy (TKE), ε, were estimated from velocity measurements acquired by a 5-beam 1 MHz Signature1000 AD2CP (Nortek AS, Norway). The ADCP was installed at 15 m water depth, looking upward. It measured flow velocities between 3.42 and 0.68 m below the lake surface with a vertical bin size of 0.02 m. Data were collected in pulse-coherent high-resolution mode as hourly bursts of 10 min duration. Each burst consisted of 1200 profiles sampled at 2 Hz.

Velocity structure function method

TKE dissipation rates ε were estimated using the velocity structure method[12] . The velocity structure function D(r) was calculated from the measured velocity along the vertical beam v(z) at the distance z from the instrument head as:

$$D(r) = \langle (v'(z) - v'(z+r))^2 \rangle$$

Here, $v'(z)$ is the along-beam velocity fluctuation at distance z, r is the depth range of the dissipation estimation, angle brackets denote time averaging over the 10-min burst. Three values of the maximum estimation range for the velocity correlation r = 0.4, 0.5, and 0.6 m were tested, covering the range recommended by Wiles at al. (2006)[12] for weakly stratified turbulence. ε was derived from the semi-empirical equation for the velocity structure function $D(r)$ along the vertical acoustic beam:

$$D(r) = Noise + C_v^2 \varepsilon^{2/3} r^{2/3}$$

Here, the constant $C_v \approx 2$[13]. The fitting constant *Noise* representing the average effect of the acoustic noise on the velocity fluctuations was used for the goodness-of-fit check, using the condition

$Noise > C_v^{-3} D^{3/2}$

Choice of r-range and stratification considerations

To discard corresponding ε values with subsequent interpolation between the neighbouring values. The discrepancy between the different estimations did not exceed 10% (not shown). The estimations based on the r = 0.4 m were adopted for the further analysis as having minimum number of bad values according to the *Noise* condition.

The ε values from the uppermost layer of 0.68-1.08 m under the lake surface were used in the further analysis.

Text S4 Calculation of turbulent kinetic energy dissipation from Acoustic Doppler velocimetry

S4.1 Evaluation of the inertial subrange method

We assessed whether the turbulent kinetic energy dissipation rate (ε) could be reliably estimated from measured flow velocity spectra using inertial subrange scaling. This method is based on Kolmogorov's law, stating that the spectral energy in flow velocity fluctuations follows a 5/3 slope, with an intercept determined by ε[14]:

$$P(k) = a \varepsilon^{2/3} k^{-5/3}$$

where P(k) is the wave number spectrum of the flow velocity ($m^3\ s^{-2}$), a is the dimensionless Kolmogorov's constant (0.49 in the direction of mean advection and 0.65 in the other direction), and k is the wave number (m^{-1}). Because we took flow measurements by maintaining the ADV in a fixed position, we used Taylor's hypothesis of frozen turbulence to convert the frequency from the power spectrum of current velocity to wavenumber: $k = 2\pi f/U$, where f is the frequency (s^{-1}) and U is the wave orbital velocity ($m\ s^{-1}$). We considered Taylor's hypothesis of frozen turbulence to be approximately valid if $\frac{u_{rms}}{U} < 0.2$, with u_{rms} being the root mean square of fluctuating velocities in direction of wave propagation[14].

Evaluating Taylor's hypothesis of frozen turbulence

To calculate U and u_{rms}, we accounted for the influence of surface waves. We identified the dominant surface wave band [illegible] from the velocity power spectral density (PSD) as the frequency range over which a distinct wave peak rose above the background turbulent spectrum. To identify the wave peak, we

screened the spectrum for local peaks, using the 'findpeaks function' of the R package 'pracma'[15]. We considered peaks with frequencies typical for lake surface waves (0.3 and 2.5 Hz[16–18]) a minimum width of 0.05 Hz and a peak height of at least 1.5 times the background turbulent spectrum. We calculated the background turbulent spectrum by linear regression of $\log_{10}$ of spectrum level vs. $\log_{10}$ of frequency across the full spectrum. As left and right boundary of the region of wave motion, we considered the frequencies where the spectrum level dropped below the background.

Wind generated waves in small lakes are often directionally spread because of short fetch length, depth-limited breaking and shoreline reflection[19,20]. We therefore determined the dominant wave propagation direction from the horizontal velocity variance structure, assuming that the wave-induced horizontal velocities are oscillatory with near-zero mean. We applied a fourth-order zero-phase Butterworth band-pass filter to the longitudinal and perpendicular horizontal velocities u and v for the wave band $[f_{min}, f_{max}]$ to obtain u_{wave} and v_{wave}, respectively. We then computed the covariance matrix of (u_{wave}, v_{wave}) and defined the principal wave axis as the leading eigenvector, which corresponds to the direction of maximum variance of the wave-induced horizontal motion. The horizontal rotation angle θ_{wave} was then obtained as in Mirshak and Kelly (2009)[21]

$$\theta_{wave} = \operatorname{atan2}(e_2, e_1)$$

where e_1 and e_2 are the components of the leading eigenvector. The velocity components were then rotated into along-wave ($u_\parallel$) and cross-wave ($u_\perp$) coordinates following standard micrometeorological procedures[22]

$$u_\parallel = u\cos(\theta_{wave}) + v\sin(\theta_{wave})$$
$$u_\perp = -u\sin(\theta_{wave}) + v\cos(\theta_{wave})$$

To account for vertical wave motions relative to the horizontal propagation direction, we applied a vertical tilt correction when the vertical oscillation amplitude exceeded a minimum threshold. Specifically, the tilt angle was defined as

$$\theta_{tilt} = \operatorname{atan2}(\sigma_w, \sigma_{u_\parallel})$$

where σ_w and $\sigma_{u_\parallel}$ are the standard deviations of the vertical component and the rotated longitudinal component along the wave propagation direction, respectively. When $|\theta_{tilt}| \geq 5°$ the velocity components were rotated in the vertical plane according to

$$u' = u_\parallel\cos(\theta_{tilt}) + w\sin(\theta_{tilt})$$

$$w' = -u_\parallel\sin(\theta_{tilt}) + w\cos(\theta_{tilt})$$

while the perpendicular horizontal component remained unchanged. If the threshold was not exceeded, the vertical tilt correction was omitted to avoid introducing noise. Because oscillatory wave orbital velocities have near-zero mean, the standard deviation provides a meaningful measure of amplitude, which justifies the use of standard deviations rather than means in defining the tilt angle.

We isolated wave orbital velocities u_{wave} and v_{wave} by applying a fourth-order zero-phase Butterworth band-pass filter to the rotated horizontal velocities u' and $u_\perp$. The effective wave orbital velocity U_{wave} was defined as the root-mean-square (RMS) magnitude of the horizontal velocity vectors:

$$U_{wave} = \sqrt{\langle u_{wave}^2 + v_{wave}^2 \rangle}$$

This vector-based definition accounts explicitly for directional spreading of the wave field and avoids underestimation that would occur if a single velocity component was used.

To isolate turbulent fluctuations, we subtracted the mean and then removed wave motions and high-frequency noise from u' and $u_\perp$ using fourth-order zero-phase Butterworth band-stop and low-pass filters

over the frequency range $[f_{min}, f_{max}]$ and the frequency associated with the characteristic length scale of the ADV measurement volume (L=14.9 mm), respectively. The remaining velocity components represent turbulence-dominated motions, u_t, v_t based on which we calculated the horizontal RMS turbulent velocity as:

$$u_{rms} = \sqrt{u_t^2 + v_t^2}$$

We evaluated Taylor's hypothesis using the ratio:

$$\frac{u_{rms}}{U_w}$$

The use of the vector RMS wave orbital velocity is particularly important in directionally spread wave fields, where single-component estimates would otherwise underestimate advection velocity and artificially inflate this ratio. To quantify the directional spreading of the wave field, we used the ratio:

$$R = \frac{S([illegible])}{S([illegible])}$$

where $R \ll 1$ indicate nearly unidirectional waves, whereas $R \approx 1$ indicate strongly multidirectional wave fields under linear wave theory[23]. Across our ADV deployments, R averaged 0.70 with a standard deviation of 0.22, indicating moderately to strongly multidirectional wave fields.

Identifying the inertial subrange

For the four deployments with approximately valid Taylor's hypothesis of Frozen turbulence, we investigated the existence of an inertial subrange. We set the low-wavenumber boundary of the inertial sub-range to the high-wavenumber boundary of the wave peak ($2\pi f_{max}/U$), or to the wavenumber ($2\pi/d$) associated to local water depth d, whichever was larger. We set the high-wavenumber boundary of the inertial sub-range to the wavenumber ($2\pi/L$) associated with the characteristic length scale of the ADV sampling volume (L=14.9 mm), or the break point in the power spectrum where the energy levels off to approach the high- wavenumber noise floor, whichever was smaller. We identified this breakpoint using the R function 'segmented'[24]. We considered the breakpoint to exist whenever a bilinear regression fit the data at least as good as a linear regression of $\log_{10}$ of spectrum level vs. $\log_{10}$ of wavenumber, for the wavenumber region bound to the left by the high-wavenumber boundary of the wave peak and to the right by the wavenumber ($2\pi f_n/U$) associated with the Nyquist frequency f_n.

Evaluation of ADV data

Overall measurement quality was high, as indicated by signal-to-noise ratios of 27±4 and inter-beam velocity correlations of 85±10% (mean±standard deviation across deployments)[14]. However, u_{rms}/U reached high levels of 0.85±1.64. Taylor's hypothesis of frozen turbulence was violated in 72% of all ADV deployments, where $\frac{u_{rms}}{U} > 2$. These measurements were therefore excluded from further analysis.

The mean flow velocity U ranged from <0.01 to 0.10 m s^{-1} and was low even for the remaining deployments with valid Taylor's hypothesis of frozen turbulence. As a result, converting frequency spectra to wavenumber space produced unrealistically large wavenumbers, exceeding the spatial resolution limit set by the ADV sampling volume. Empirical velocity wave-number spectra diverged clearly from theoretical Nasmyth universal spectra, with less of a decade energy drop in the wavenumber region with a spectral slope of -5/3 (Supplementary Fig. S12). Consequently, the inertial subrange could not be resolved with sufficient accuracy, and estimation of ε using the inertial subrange method was not possible.

S4.2 Calculation of turbulent kinetic energy dissipation using large-eddy scaling

In a system where production of turbulence equals its dissipation, a local balance occurs, and the dissipation rate ε can be determined by the rate of energy input at the largest scales of the turbulent motion. Near the lake surface, turbulent energy is injected directly by wind and wave processes and dissipated over short spatial and temporal scales, so that the turbulence measured at a given location primarily reflects local energy input rather than remote transport. Assuming turbulence is fully developed over the depth, ε can be scaled using the characteristic turbulent velocity U and the characteristic length scale of the energy-containing eddies L[25]:

$$\varepsilon = C_\varepsilon U^3 / L$$

where C_ε is a dimensionless flow specific dissipation constant that typically ranges from approximately 0.5 to 1.5[26], and here we assumed $C_\varepsilon = 1$ owing to a lack of empirical estimates from lakes. We considered L to be water depth and $U = u_{rms}$ assuming that u_{rms} is dominated by turbulence, after wave motions and high frequency noise were removed as described in Supplementary Text S2.1. This RMS-based large-eddy estimate of ε quantifies the total turbulent energy cascade that is relevant for surface renewal processes governing air–water gas exchange.

Text S5 Bubble gas exchange modelling

In the calculation of microbubble-mediated CH_4 flux, we assumed that the microbubbles were in equilibrium with the surrounding water at the time they reached the water surface. To justify this assumption, we used a physical model of bubble-water gas exchange. The bubble equilibration time (s) was calculated as[27]

$$T_g = [illegible]$$

where r is bubble radius (m), $\alpha = [illegible]$ is Ostwald solubility (unitless)[8], β is Bunsen solubility (mol m^{-3} Pa^{-1}) derived from water temperature (T_w) and salinity[7] (calculated from electrical conductivity (EC), water temperature and air pressure (P_{atm}))[28], and j is bubble-water gas transfer velocity for dirty bubbles (m s^{-1}). Given the large variability among published parameterizations of j we considered both a low estimate[27],

$$j = [illegible]$$

and a high estimate[29]

$$j = [illegible]$$

where g is gravity acceleration (9.81 m s^{-2}), ν is kinematic viscosity derived from temperature (m^2 s^{-1})[30], $D = \frac{\nu}{Sc}$ is diffusivity, Sc is Schmidt number derived from temperature and salinity, and V_b is bubble rise velocity. To account for size-dependency of published parameterizations of V_b we modelled V_b for $r < 600$ µm as[5]

$$V_b = \frac{2 r^2 g \rho}{9 \nu} [illegible]$$

where $y = [illegible]$ is a shape correction factor (unitless), $X = [illegible]$ and ρ is water density (Kg m^{-3}) derived from water temperature and salinity[31]. The rise velocity of bubbles with $r \geq 600$ µm was modelled as[32]

$$V_b = [illegible]$$

For reference, we also calculated an idealized bubble residence time T as

$$T = z / V_b$$

where z=0.05 m was the approximate depth of microbubble measurements in this study. Hence, T represents the time for a bubble to rise from 5 cm depth to the surface under the first-order assumption that bubble–flow interactions are negligible

We calculated T and T_g for a range of bubbles with 9–44 µm radius, assuming T=18.7°C, EC=251 µS cm^{-1}, P_{atm}=1006 hPa), i.e. mean values across all measurements. Model output is shown in Supplementary Figure S14

Supplementary figures

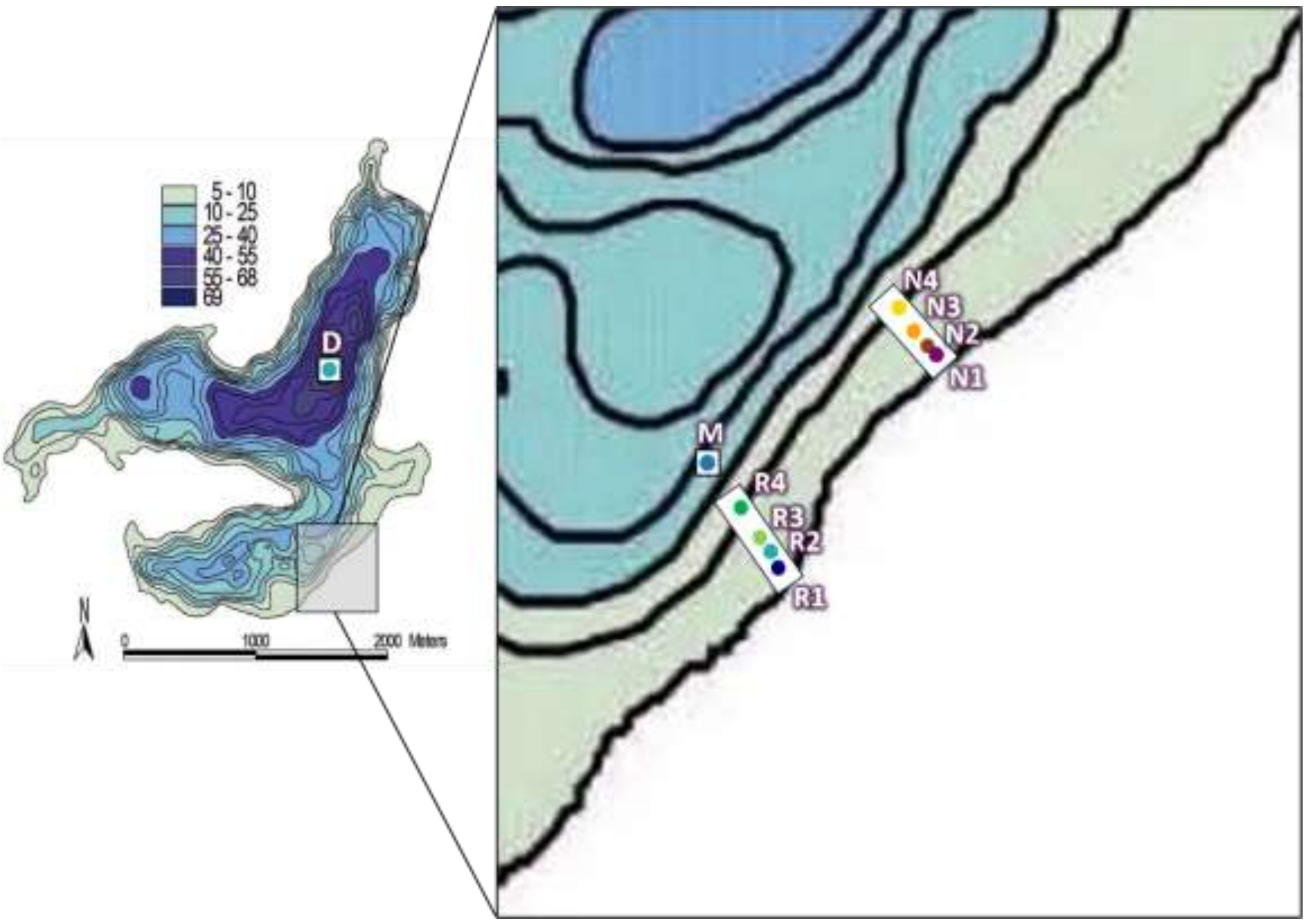

Fig. S1. Bathymetric map of Lake Stechlin. Units of depth contours are in meters. Circles show sampling sites, including the offshore mid-lake station (M), the deep-basin station (D), and two littoral transects: a macrophyte-rich, reed-dominated transect (R1–R4) and a transect without reed and with sparse macrophyte cover (N1–N4). Modified from McGinnis et al. (2015)[33].

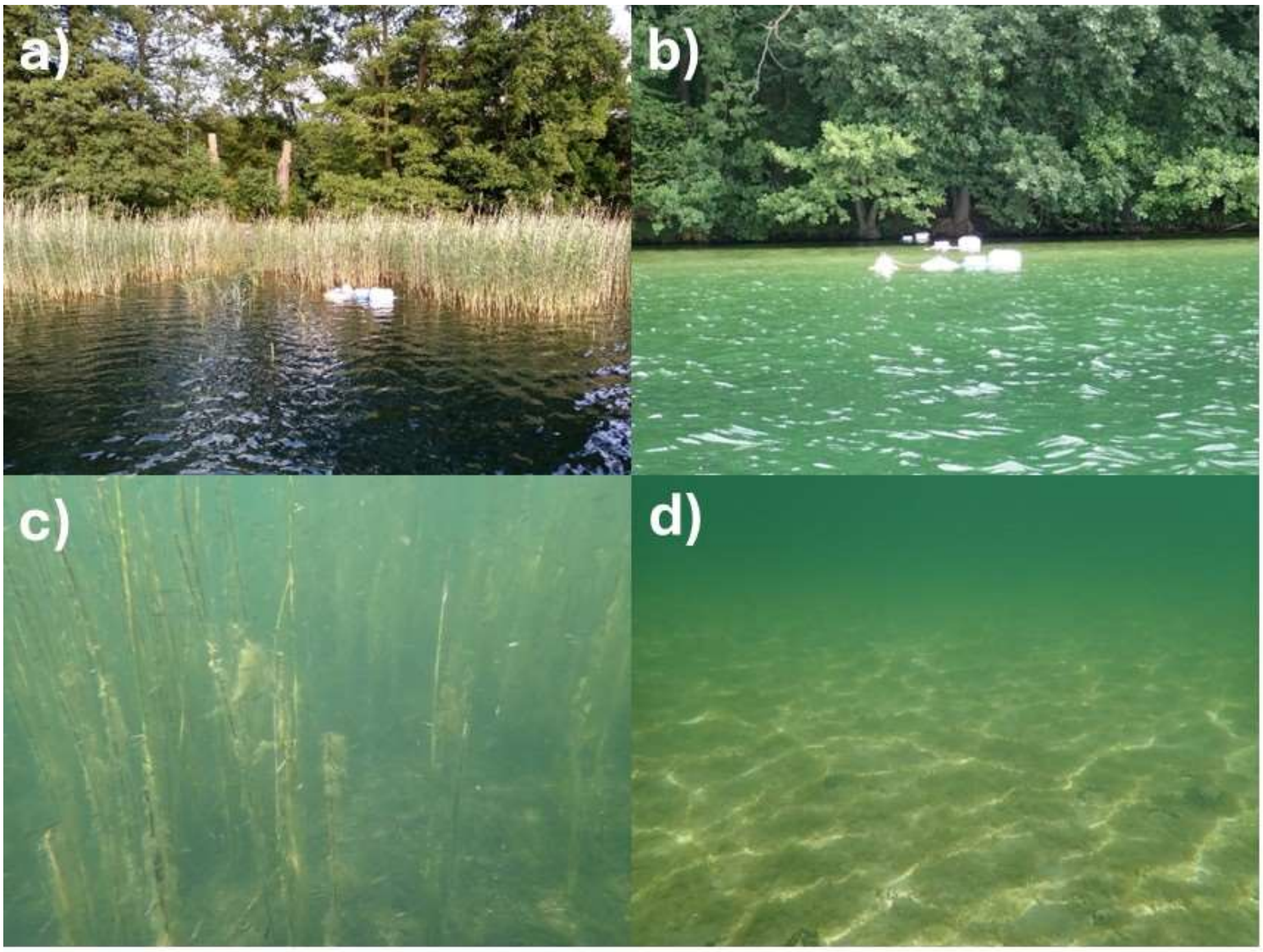

Fig. S2. Photographs of the littoral transects dominated by reed and with dense macrophyte cover (a, c) and free of reed and sparse macrophyte cover (b, d). Buoys mark the sampling sites R1-R3 and N1-N3, respectively. Photo is taken approximately from site R4 and N4, respectively.

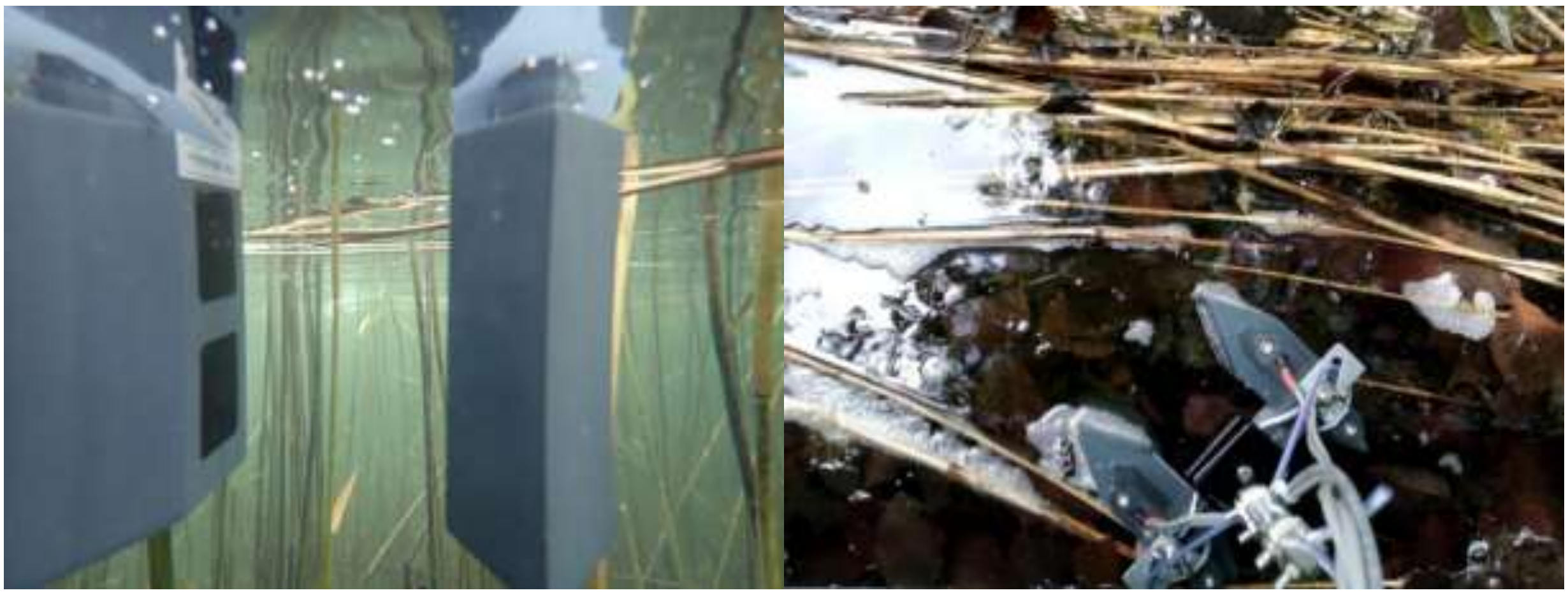

Fig. S3. Hydrophones as part of the Acoustic Bubble spectrometer (ABS). Note the foam floating on the water surface, indicating bubble entrainment and stabilization through surfactants stemming form e.g. decaying plankton and other organic material.

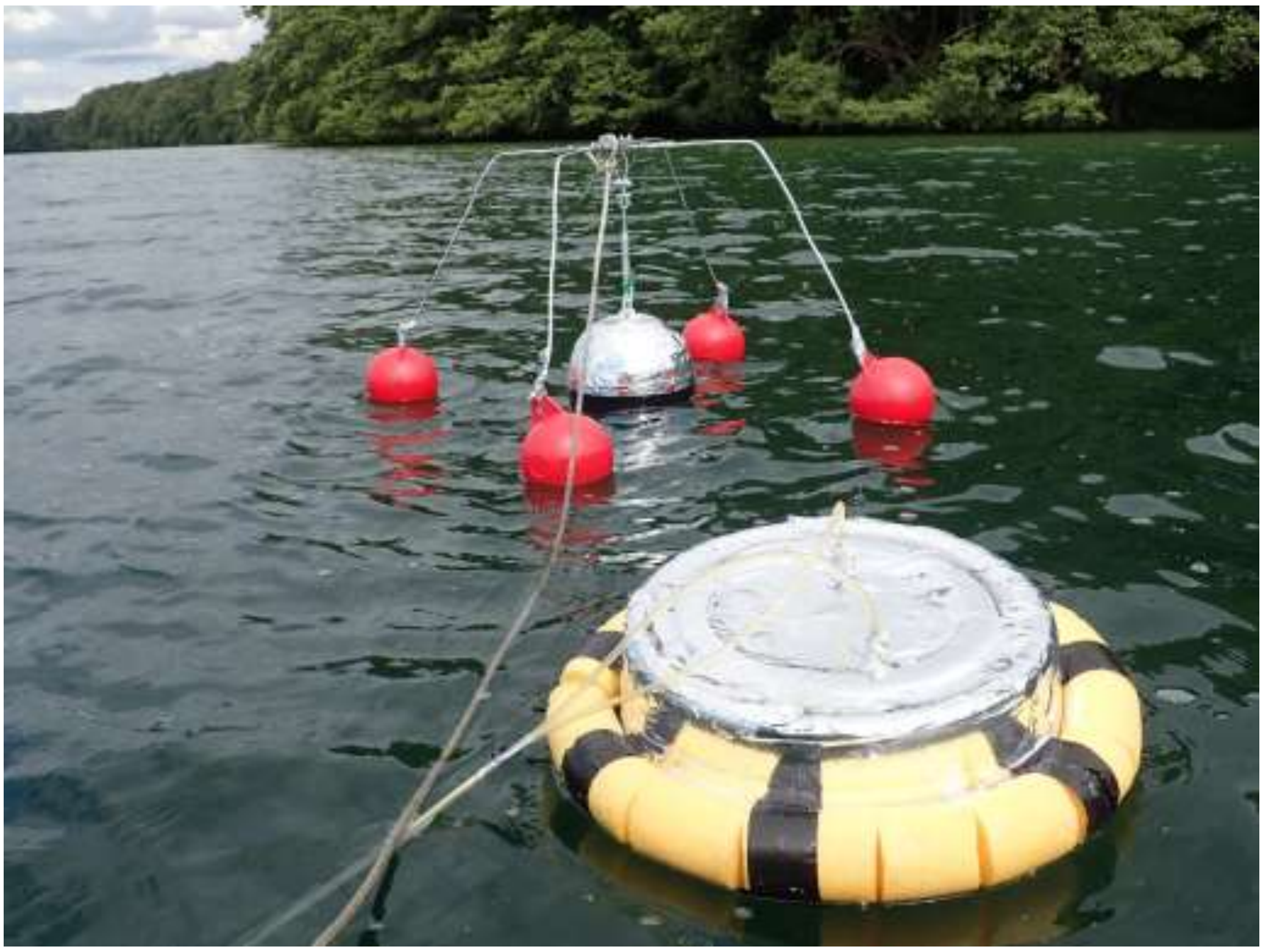

Fig. S4. Flux chambers deployed in this study: Floating chamber (front) and flying chamber (back).

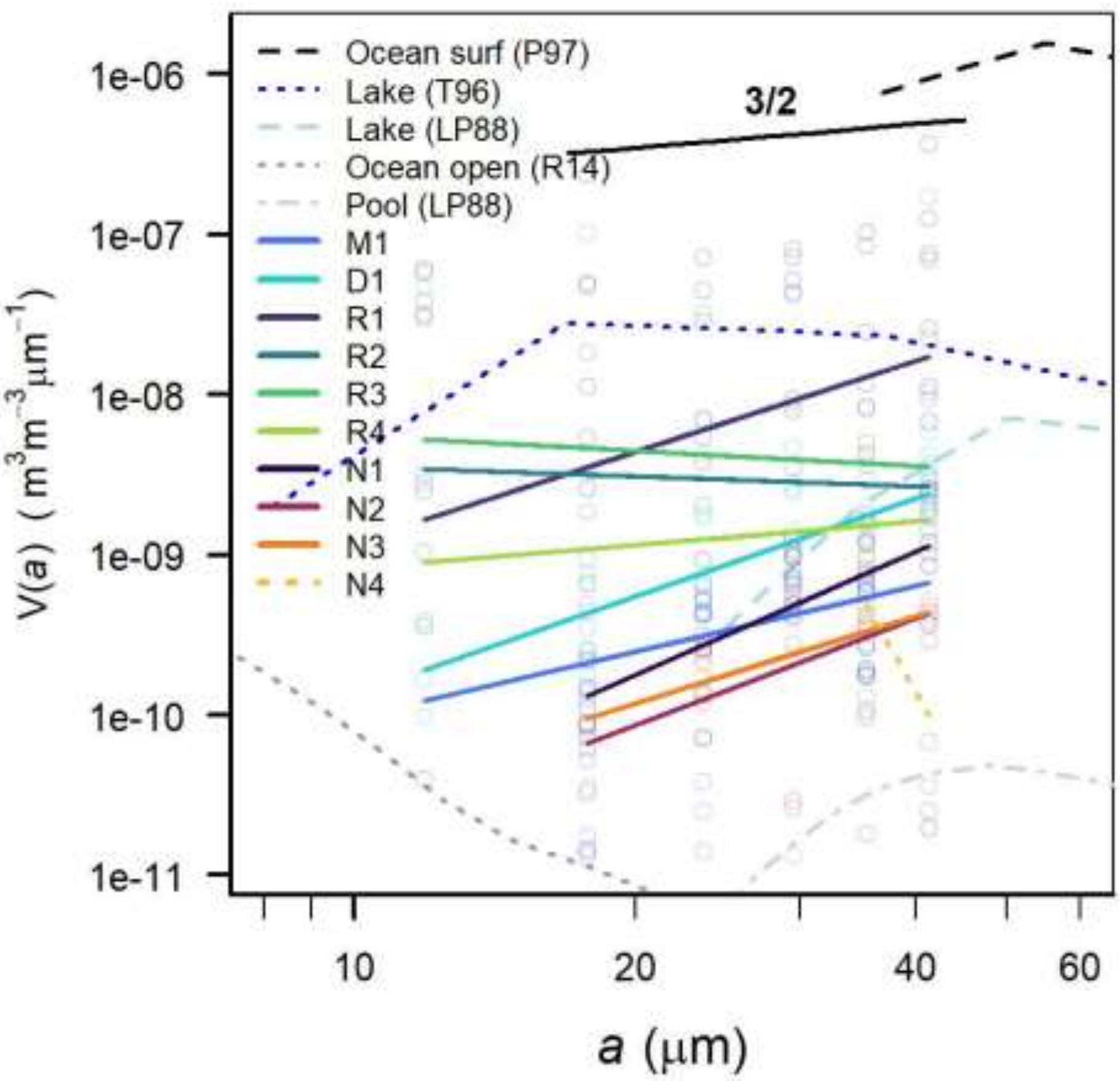

Fig. S5. Microbubble void fraction as function of bubble radius (*a*). Circles show individual observations while colored solid lines show least-squares linear regressions in log-log scale. Color code of circles and lines represents sampling sites (M; mid lake, D; deepest point, R1-R4; littoral reed transect and N1-N4; littoral non-reed transect). Yellow dashed line for site N4 represents value close to the detection limit of instrument. The black thick line shows a theoretical spectral slope of 3/2 that would be expected if wave-induced entrainment was the main microbubble source[34]. Literature data is from Phelps et al. (1997)[35] (P97), Trevorrow (1996)[36] (T96), Ling and Pao (1988)[37] (LP88) and Randolph et al. (2014)[38] (R14).

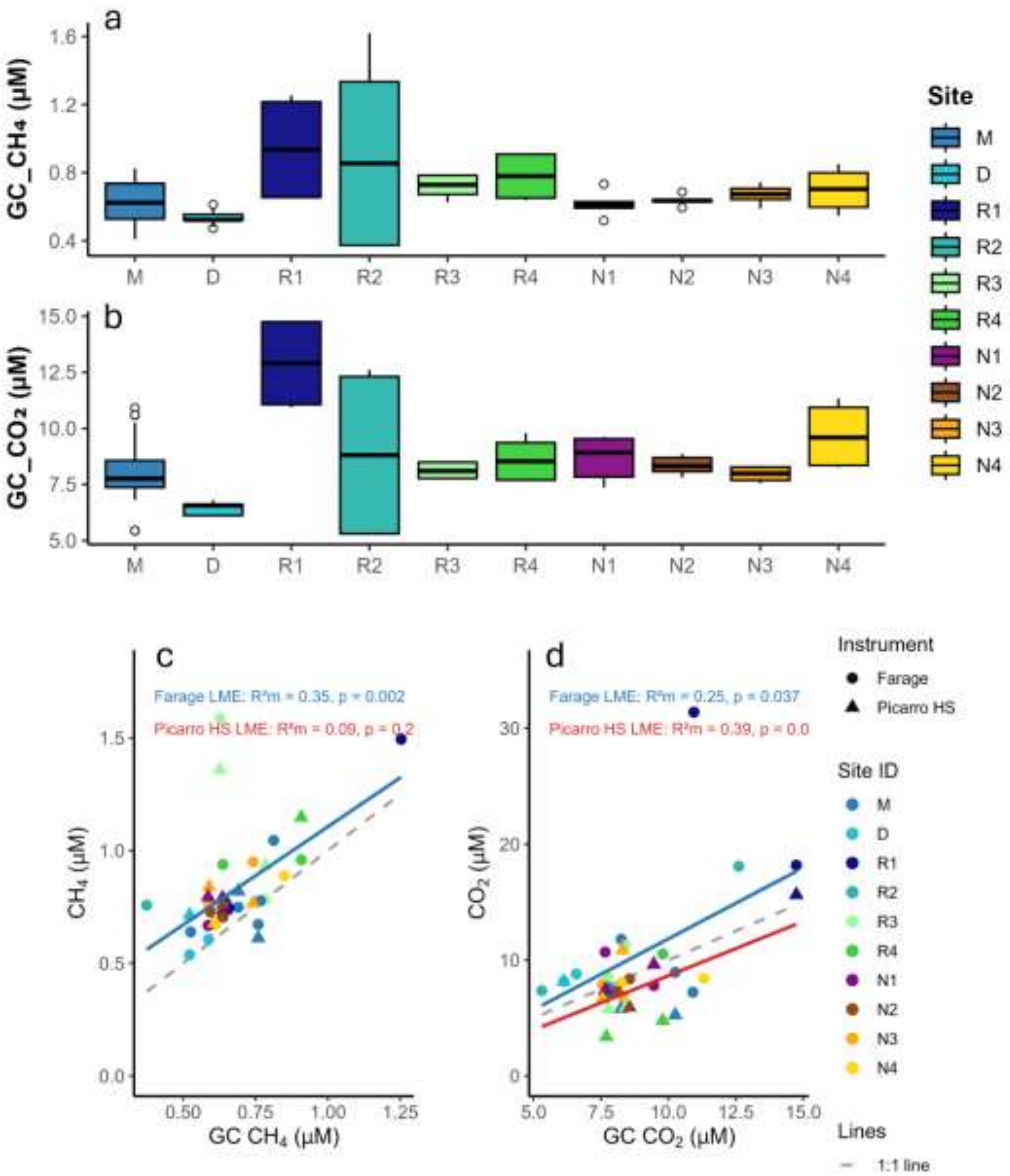

Fig. S6. Concentration of dissolved CH_4 (a) and CO_2 (b) across sites based of GC method (a). The boxes represent the interquartile range, the whiskers extend to 1.5 times the interquartile range, circles represent outliers, and the black lines inside the box indicate the median. Scatter plot comparing Farage (circles) and Picarro HS (triangles) concentration measurements (vertical axes) with GC method (horizontal axes) for CH_4 (c) and CO_2 (d), with points colored by site. The lines show linear mixed effects model (LME) predictions, when slopes were significant ($p<0.05$).

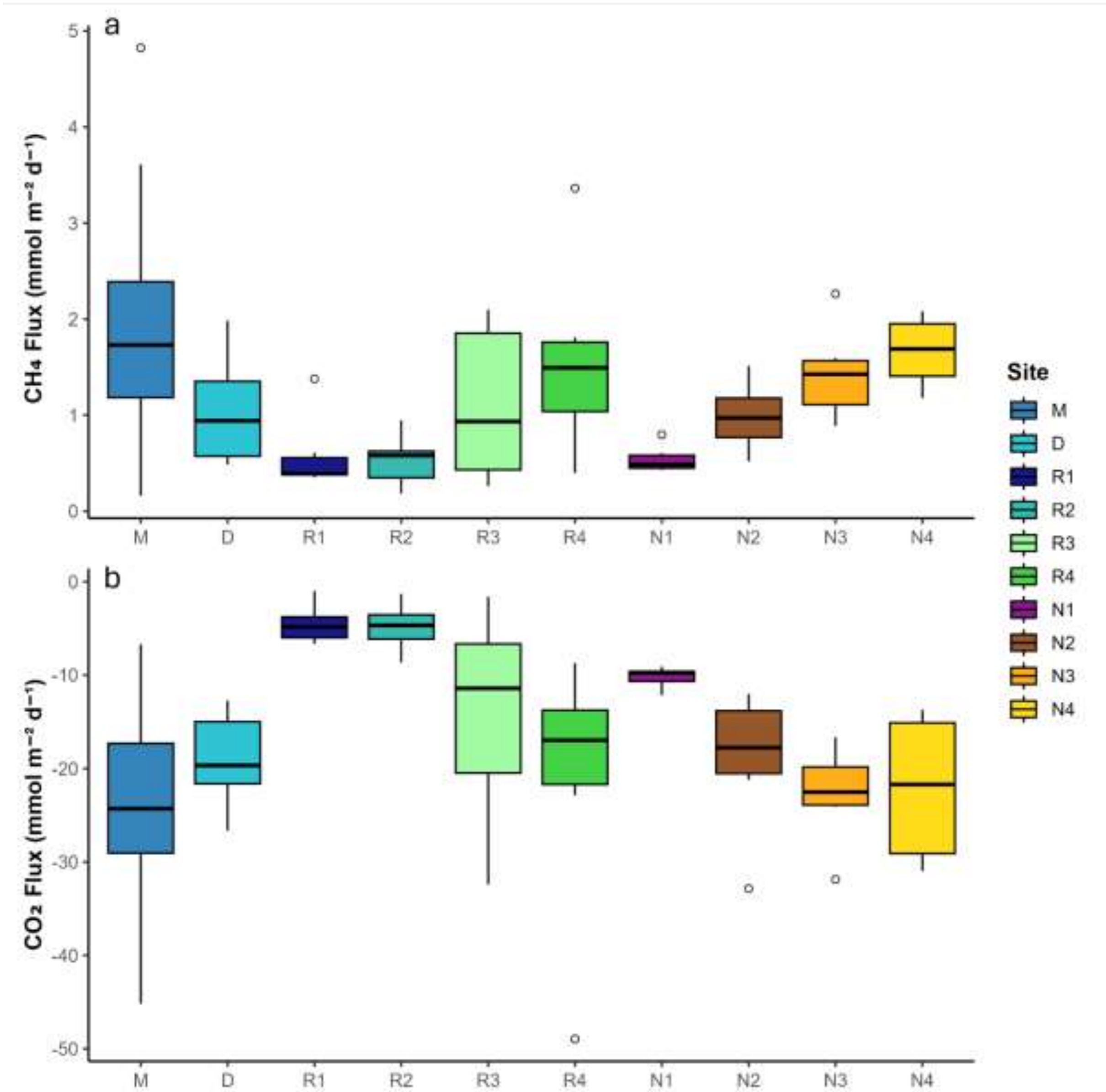

Fig. S7. CH_4 (a) and CO_2 (b) flux distribution across sites based on floating chamber. The boxes represent the interquartile range, the whiskers extend to 1.5 times the interquartile range, circles represent outliers, and the black lines inside the box indicate the median.

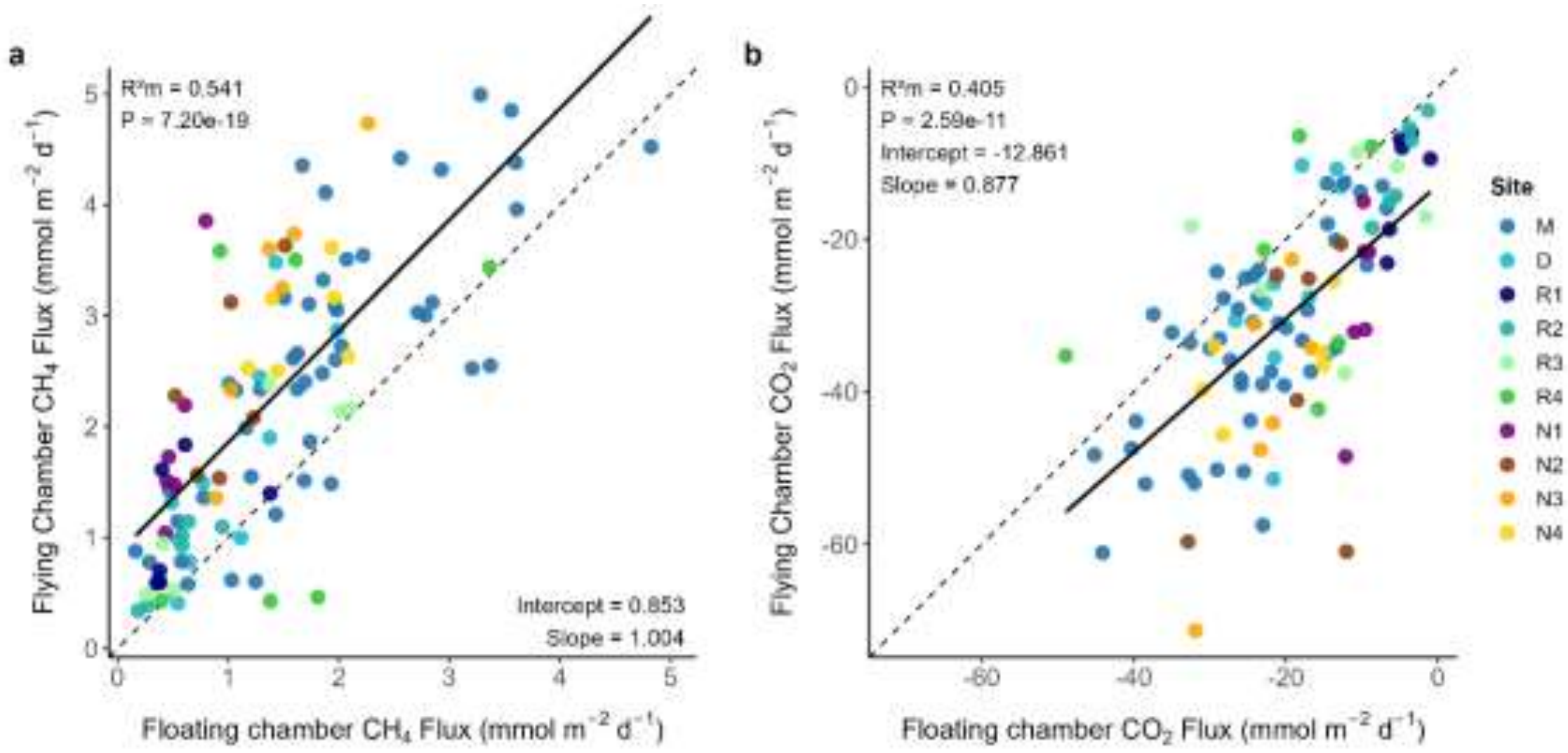

Fig. S8. Relationship between floating chamber and flying chamber fluxes of CH_4 (a) and CO_2 (b) across all sites. Each point represents a paired flux measurement. Solid lines show the fixed-effect fit from a linear mixed-effects model with random intercepts by site, and dashed lines indicate the 1:1 line.

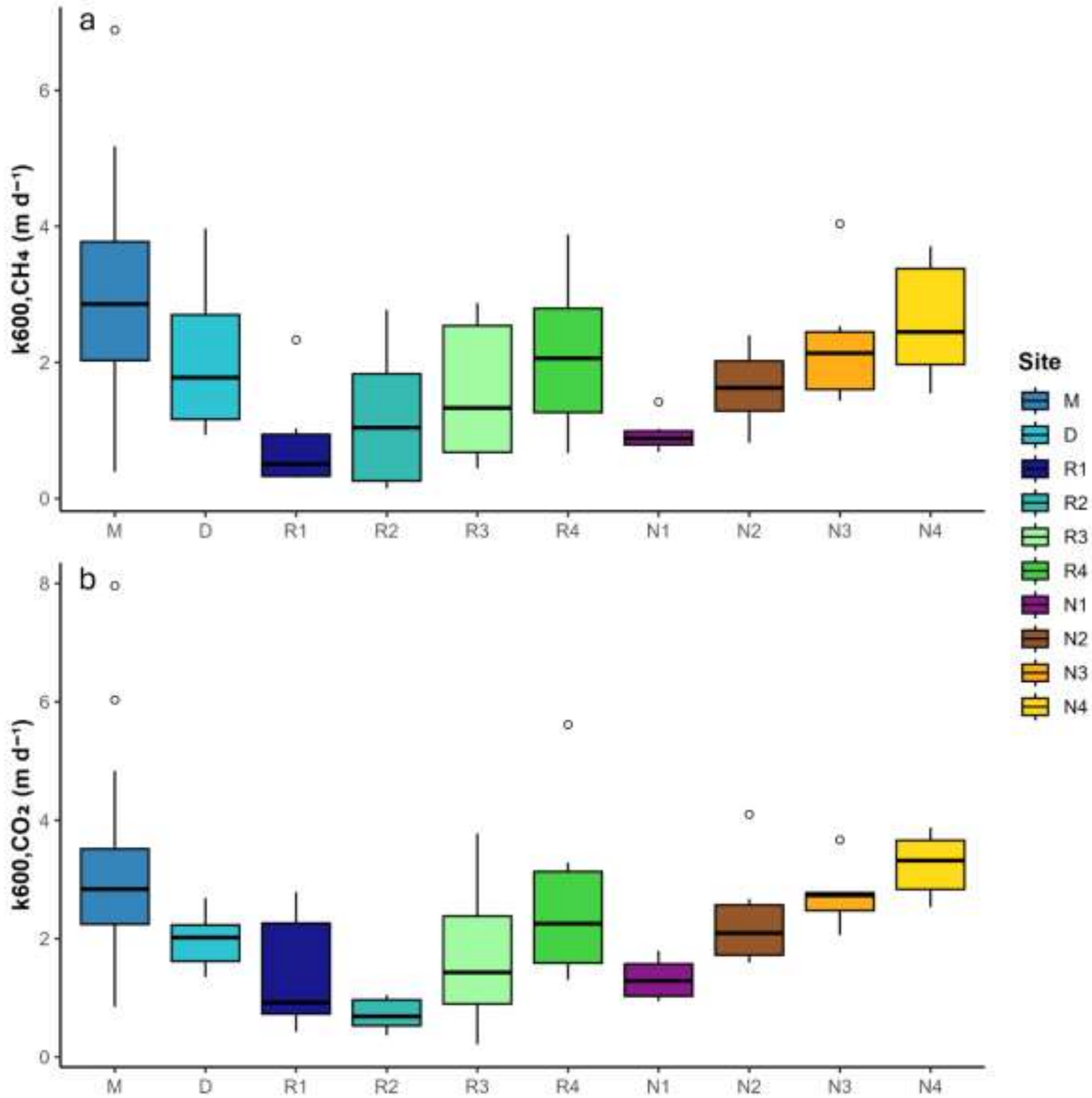

Fig. S9. Distributions of gas transfer velocities (k_{600}) for CH_4 (a) and CO_2 (b) across the sites estimated from floating chamber flux data. The boxes represent the interquartile range, the whiskers extend to 1.5 times the interquartile range, circles represent outliers, and the black lines inside the box indicate the median.

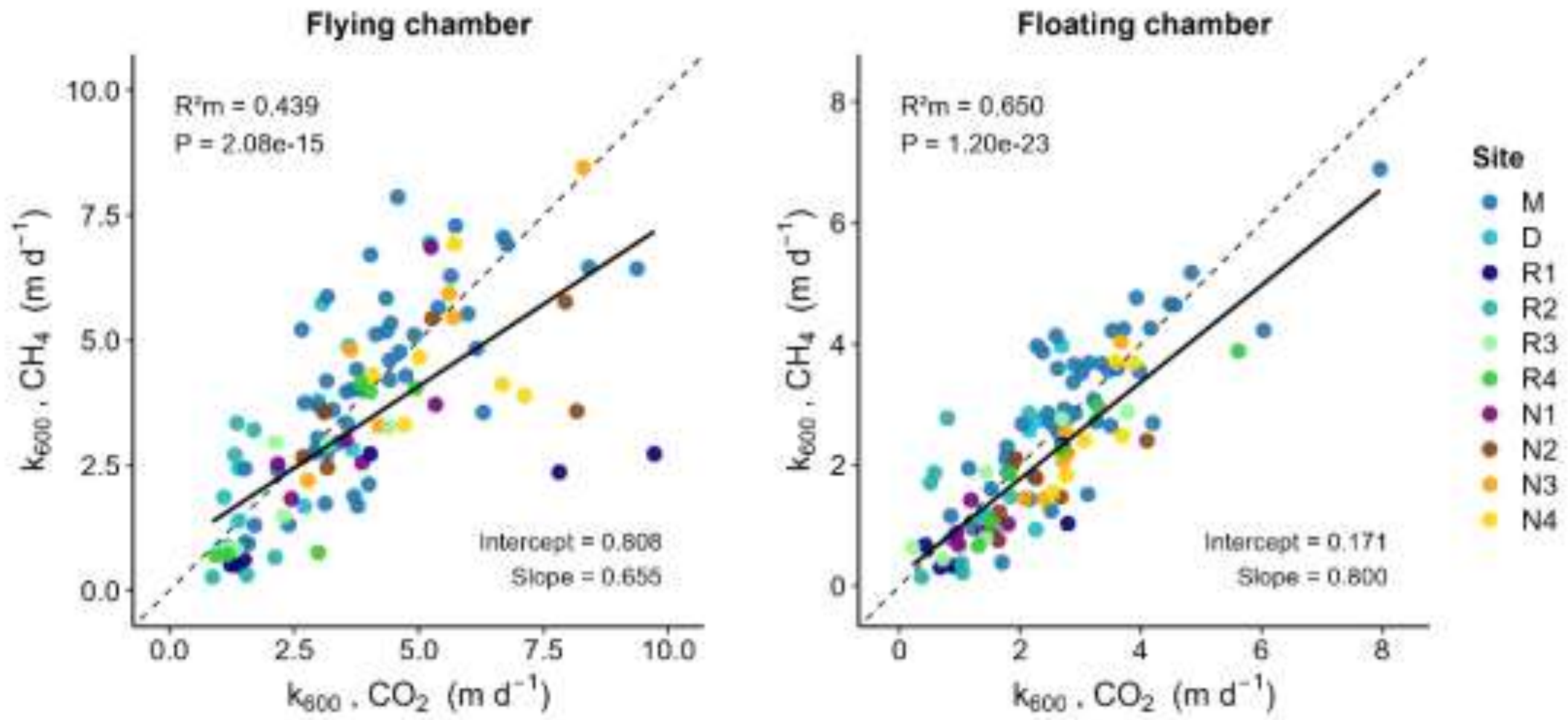

Fig. S10. Relationship between $k_{600,CH4}$ and $k_{600,CO2}$ obtained from flying chamber (left panel) and floating chamber (right panel) fluxes. Solid lines show the fixed-effect fit from a linear mixed-effects model with random intercepts by site, and dashed lines indicate the 1:1 line.

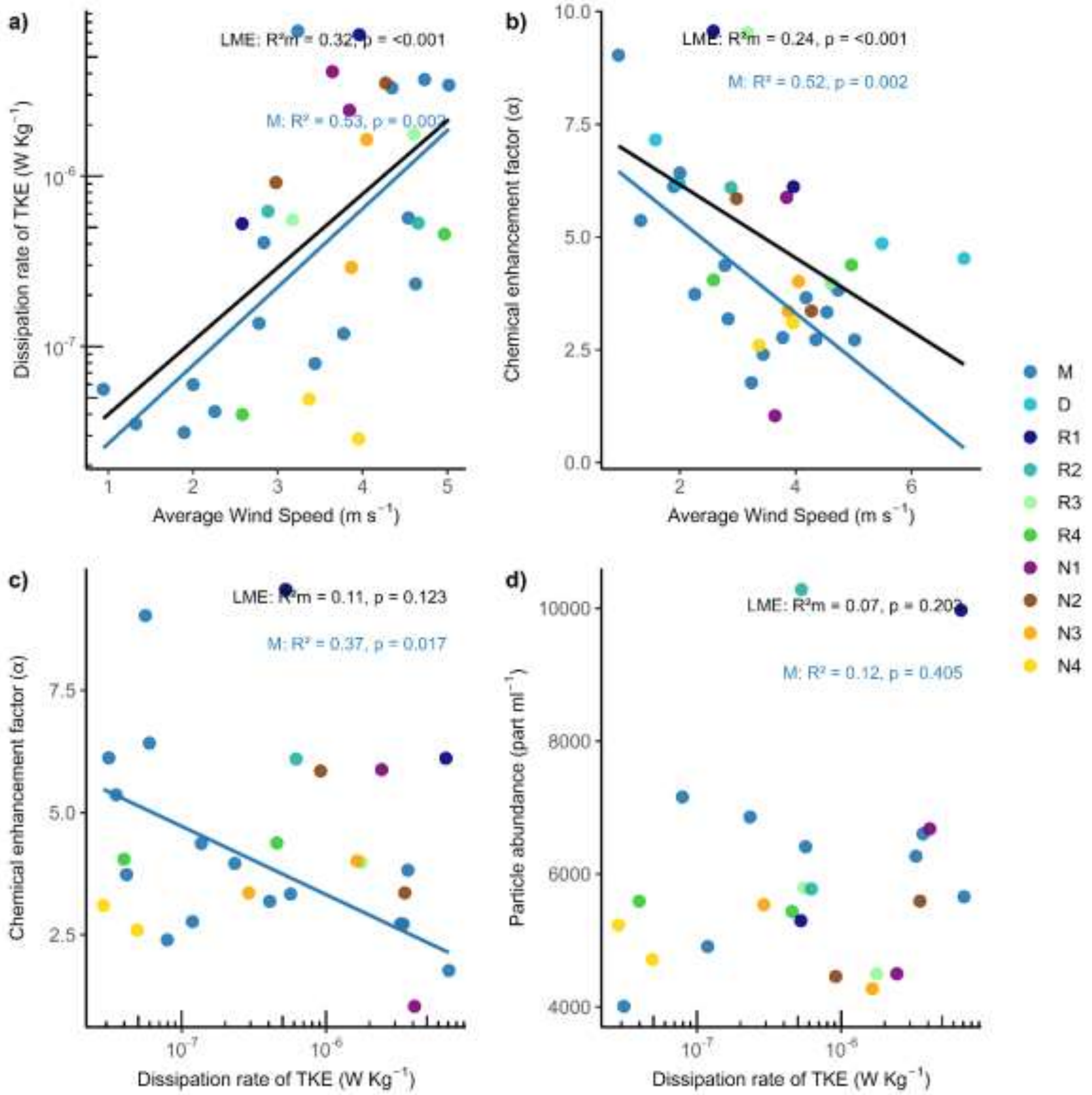

Fig. S11. Relationship between (a) average wind speed and dissipation rate of TKE (b) average wind speed and chemical enhancement factor (α), (c) dissipation rate of TKE and α and (d) α and particle abundance. Points are colored by sampling site; solid lines indicate significant fits from a mixed-effects model (all sites; black) and the blue line show linear model fit only for the M site.

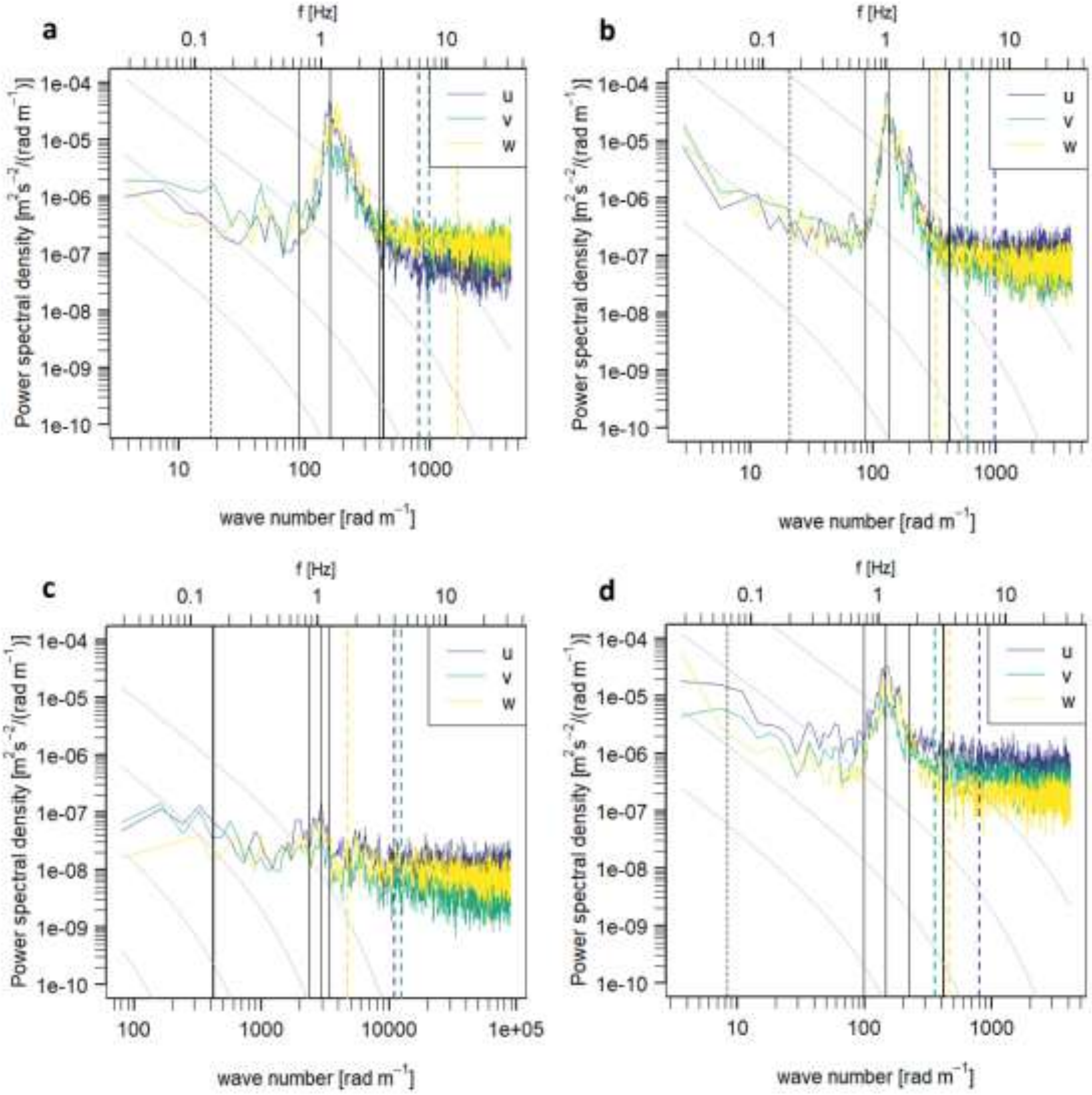

Fig. S12. Selected examples of flow velocity – wavenumber spectra derived from Acoustic Doppler Velocimetry. Measurements are from (A) Site N1, 16 June 2024, (B) Site R1, 13 June 2024, (C) Site R3, 14 June, (D) Site N2, 16 June. Blue, turquoise and yellow thin solid lines show spectra of flow velocities in longitudinal, perpendicular and vertical direction (u, v, w), respectively. Thick dashed lines, coloured accordingly, show the breakpoints in the spectra that approximate the onset of the noise floor. The black dashed thin vertical line shows the wavenumber associated with local water depth. Black thin solid vertical lines show the maximum and the left and right boundary of the surface wave peak. The black thick solid vertical line shows the wavenumber associated with the spatial resolution limit set by the ADV sampling volume. Grey lines show the theoretical Nasmyth universal spectrum for ε of 10^{-2}, 10^{-4}, 10^{-6} and 10^{-8} m^2 s^{-3} following Oakey (1982)[39].

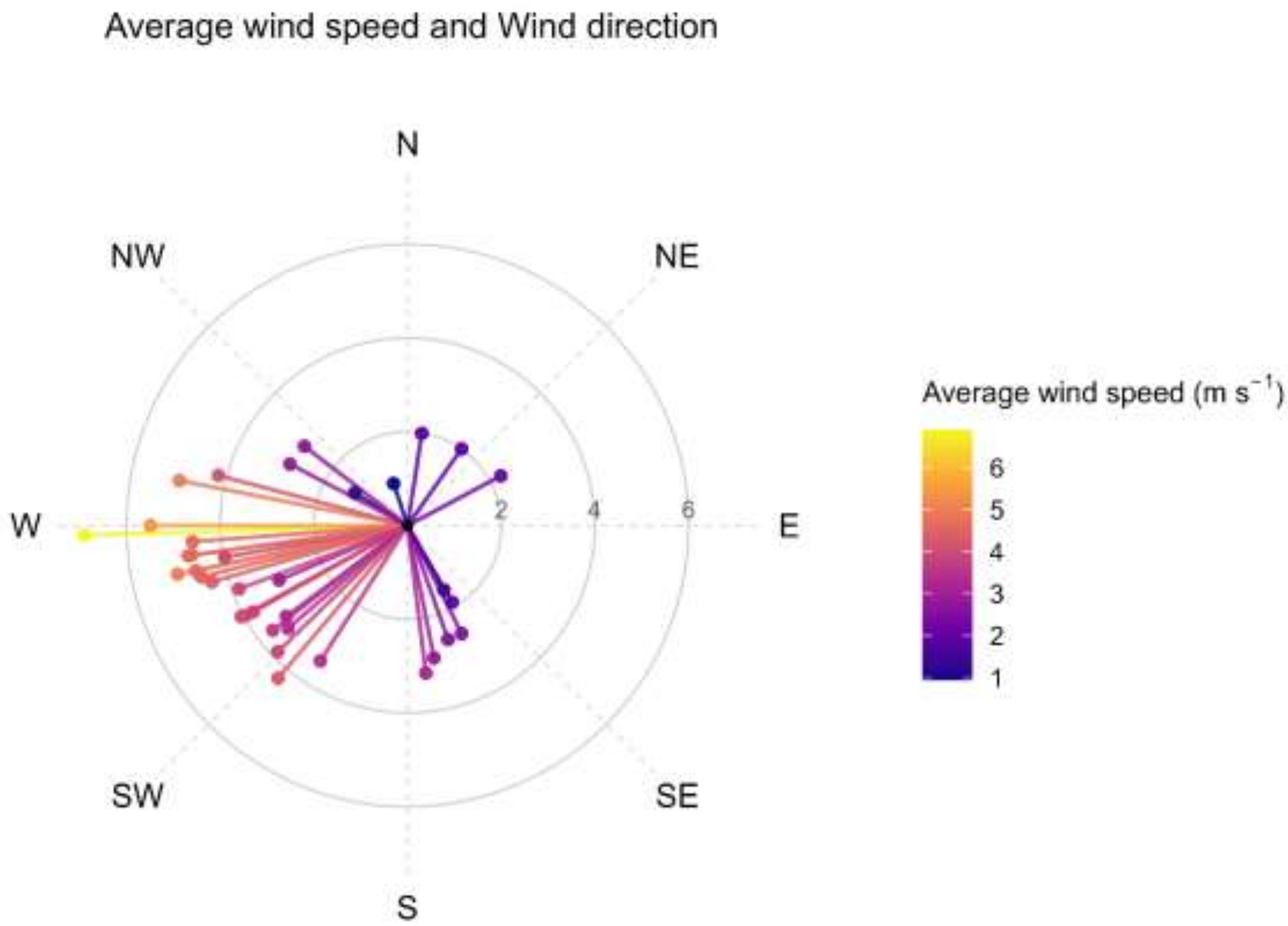

Fig. S13. Average wind speed at each sampling hour and wind direction.

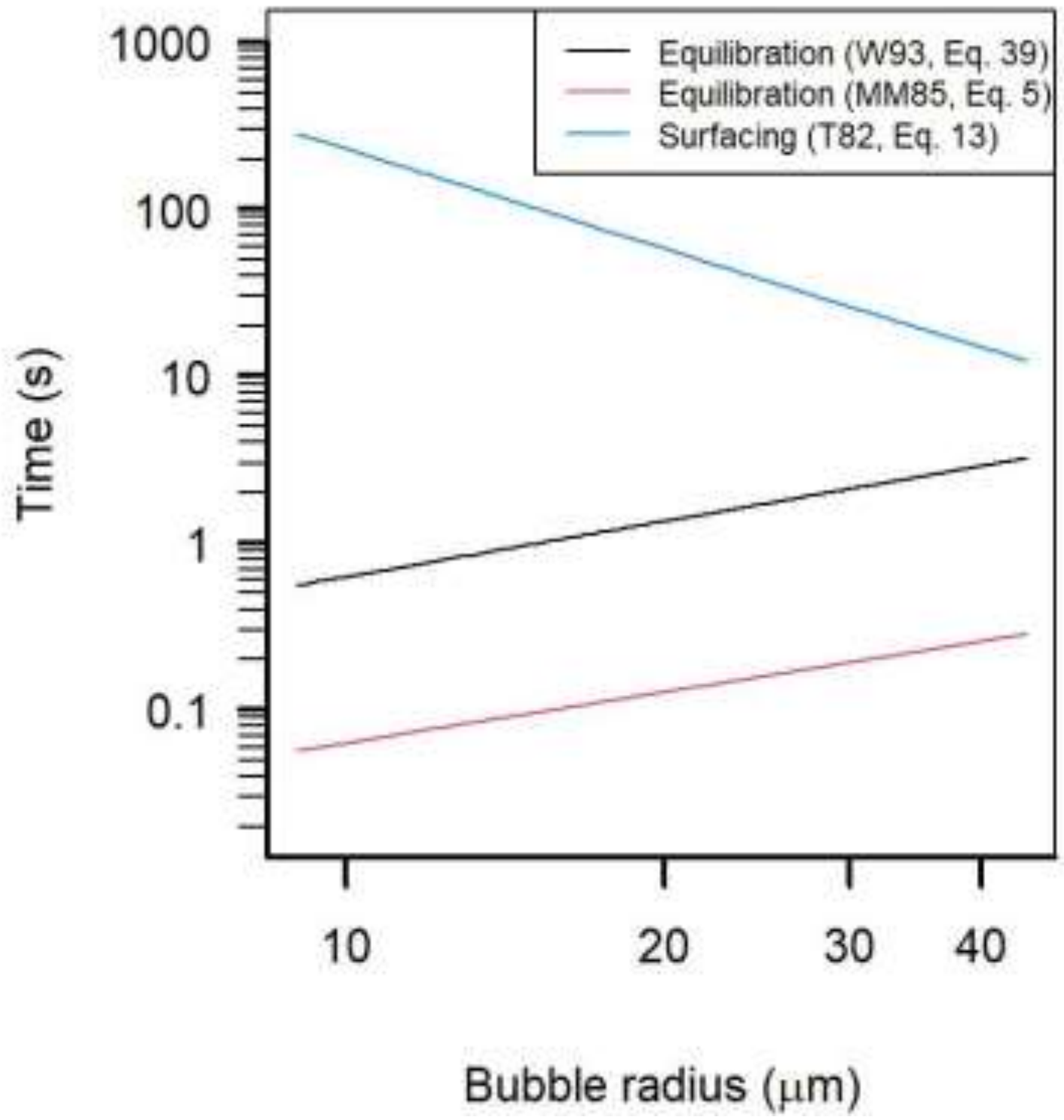

Fig. S14. Modelled time scales for bubble equilibration and residence as a function of bubble radius. Solid lines show the equilibration time based on the low and high estimates from Woolf et al. (1993)[27] (W93, Eq. 39) and Memery and Merlivat (1985)[29] (MM85, Eq. 13), respectively.

Supplementary Tables

Table S1. Summary (mean ± SD) of environmental, physical, and aqueous chemical parameters measured at each site. For Dissipation rate of TKE (ε) and microbubble void fraction (V), were log10-transformed and displayed on the log10 scale. Wind speed relates to 10 m above ground.

Site ID	Water depth (m)	Distance to shore (m)	T_{water} (°C)	Wind speed (m s^{-1})	Oxygen saturation (%)	pH	Electrical conductivity (µS cm^{-1})	CH_4 (µM)	CO_2 (µM)	CH_4 flux (mmol m^{-2} d^{-1})	CO_2 flux (mmol m^{-2} d^{-1})	k_{600} CH_4 (m d^{-1})	k_{600} CO_2 (m d^{-1})	k_{600} ratio (CH_4/CO_2)	$log_{10}(ε)$	$log_{10}(V)$
D	68		19.40 ± 0.31	3.99 ± 2.62	112.41 ± 0.35	8.77 ± 0.02	250.50 ± 9.48	0.53 ± 0.04	6.43 ± 0.28	1.02 ± 0.50	-18.91 ± 4.61	2.00 ± 1.01	1.96 ± 0.43	0.99 ± 0.33	NA	-8.41 ± 0.36
M	15		19.24 ± 0.79	3.24 ± 1.29	110.57 ± 1.87	8.68 ± 0.05	251.51 ± 5.62	0.63 ± 0.13	8.04 ± 1.25	1.85 ± 1.02	-24.22 ± 9.62	2.97 ± 1.33	2.98 ± 1.31	1.04 ± 0.31	-6.95 ± 0.83	-8.86 ± 0.60
N1	0.35	4	18.95 ± 0.82	3.74 ± 0.14	110.35 ± 3.66	8.66 ± 0.06	254.00 ± 6.57	0.62 ± 0.07	8.67 ± 1.02	0.54 ± 0.14	-10.21 ± 1.13	0.94 ± 0.26	1.32 ± 0.35	0.75 ± 0.27	-5.50 ± 0.16	-8.74 ± 0.43
N2	0.75	12	19.20 ± 1.20	3.62 ± 0.92	112.91 ± 7.76	8.67 ± 0.01	249.00 ± 8.76	0.64 ± 0.03	8.35 ± 0.42	0.99 ± 0.36	-19.06 ± 7.60	1.63 ± 0.58	2.37 ± 0.94	0.71 ± 0.22	-5.75 ± 0.41	-9.01 ± 0.72
N3	1.5	28	19.25 ± 0.05	3.96 ± 0.13	114.34 ± 1.03	8.69 ± 0.03	244.00 ± 2.74	0.67 ± 0.05	7.97 ± 0.35	1.44 ± 0.49	-22.81 ± 5.22	2.29 ± 0.96	2.73 ± 0.54	0.81 ± 0.18	-6.16 ± 0.53	-8.99 ± 0.36
N4	5	50	19.30 ± 0.00	3.66 ± 0.41	110.52 ± 1.14	8.61 ± 0.05	244.00 ± 2.19	0.70 ± 0.13	9.68 ± 1.50	1.67 ± 0.37	-22.09 ± 8.22	2.61 ± 0.91	3.25 ± 0.54	0.79 ± 0.17	-6.47 ± 0.77	-9.66 ± 0.48
R1	0.3	5	18.15 ± 0.60	3.27 ± 0.98	105.84 ± 2.86	8.51 ± 0.06	255.00 ± 1.10	0.94 ± 0.31	12.88 ± 2.04	0.58 ± 0.40	-4.52 ± 2.09	0.83 ± 0.79	1.41 ± 1.05	0.66 ± 0.49	-5.72 ± 0.78	-7.48 ± 1.26
R2	0.75	15	18.45 ± 0.38	3.77 ± 1.25	105.84 ± 0.33	8.60 ± 0.10	253.50 ± 8.22	0.90 ± 0.59	8.85 ± 3.89	0.53 ± 0.27	-4.84 ± 2.56	0.42 ± 0.35	0.78 ± 0.32	0.57 ± 0.50	-6.24 ± 0.05	-8.22 ± 1.11
R3	1.5	20	18.75 ± 0.27	3.89 ± 1.01	110.29 ± 0.44	8.68 ± 0.02	254.50 ± 4.93	0.72 ± 0.07	8.13 ± 0.39	1.11 ± 0.83	-14.24 ± 11.55	1.56 ± 1.09	1.71 ± 1.31	1.20 ± 0.89	-6.01 ± 0.36	-7.89 ± 1.12
R4	5	55	18.75 ± 0.05	3.77 ± 1.68	110.84 ± 2.37	8.66 ± 0.05	256.50 ± 0.55	0.78 ± 0.14	8.60 ± 1.00	1.58 ± 1.01	-21.26 ± 14.38	2.12 ± 1.19	2.70 ± 1.61	0.78 ± 0.18	-7.01 ± 1.06	-8.44 ± 0.99

Table S2a. Summary of linear mixed effect model for $k_{600,CH4}$ as predicted and $k_{600,CO2}$ as predictor variable across sampling sites. Sampling site was included as a random intercept.

LME Model	Fixed effects	Estimate	Std. Error	t-value	p-value	R^2_m
$k_{600,CH4}$ ~ $k_{600,CO2}$	Intercept	0.043	0.167	0.255	7.999e-01	0.693
	$K_{600,CO2}$	0.828	0.056	14.678	9.567e-27	

Table S2b. Summary of linear model for $k_{600,CH4}$ as predicted and $k_{600,CO2}$ as predictor for the M site.

LM Model	Predictor	Estimate	Std. Error	t-value	p-value	R^2
$k_{600,CH4}$ ~ $k_{600,CO2}$	Intercept	0.605	0.281	2.153	3.683e-02	0.667
	$K_{600,CO2}$	0.812	0.086	9.386	4.547e-12	

Table S3a: Effect of microbubble void fraction, physical and environmental variables on k_{600} ratios across sampling sites as shown by linear Mixed Effect (LME) Model. The fixed effects include mean microbubble void fraction (V; scaled by 10^7 to improve numerical readability), the TKE dissipation rate (ε), the hourly average wind speed (U), and the chemical enhancement factor (α). Sampling site was included as a random intercept. R^2_m is marginal coefficient of determination, AIC is Akaikes Information Criterion.

LME Model	Fixed effects	Estimate	Std. Error	t-value	p-value	R^2m	AIC
K_{600} ratio ~ log10(V)	Intercept	1.525	0.44	3.462	0.001	0.06	16.3
	log10(V)	0.086	0.055	1.554	0.129		
K_{600} ratio ~ log10 (ε)	Intercept	0.931	0.413	2.25	0.032	0.002	16.1
	log10 (ε)	0.016	0.065	0.253	0.801		
K_{600} ratio ~ U	Intercept	0.384	0.117	3.26	0.0024	0.30	3.83
	U	0.124	0.028	4.37	< 0.001		
K_{600} ratio ~ α	Intercept	1.039	0.117	8.866	< 0.001	0.12	10.4
	α	-0.047	0.021	-2.244	0.031		
K_{600} ratio ~ U + α	Intercept	0.416	0.199	2.08	0.044	0.27	2.00
	U	0.111	0.031	3.57	< 0.001		
	α	-0.004	0.021	-0.230	0.818		

Table S3b: Effect of microbubble void fraction, physical and environmental variables on k_{600} ratios for the M sites in as shown by linear model. The predictors include mean microbubble void fraction (V; scaled by 10^7 to improve numerical readability), the TKE dissipation rate (ε), the hourly average wind speed (U) and the chemical enhancement factor (α). R^2 is coefficient of determination.

Predicted	Term	Estimate	Std. Error	t-value	p-value	R^2	AIC
K_{600} ratio	Intercept	1.449	0.949	1.527	0.149	0.015	7.74
	log10(V)	0.053	0.116	0.456	0.655		
	Intercept	1.48	0.597	2.47	0.027	0.045	8.13
	log10 (ε)	0.07	0.090	0.779	0.449		

	Intercept	0.631	0.156	4.04	0.001		1.47
	U	0.118	0.044	2.65	0.01	0.33	
	Intercept	1.1	0.17	6.472	< 0.001		7.66
	α	-0.02	0.038	-0.529	0.605	0.02	

Table S4a: Effects of physical and chemical parameters on the microbubble void fraction shown by Mixed effect modelling. The fixed effects are hourly mean wind speed (U), the TKE dissipation rate (ε), and the particle abundance (P_A). Sampling location was included as a random intercept. R^2_m is marginal coefficient of determination, AIC is Akaikes Information Criterion.

LME Model	Fixed effects	Mean	Std. Error	t-value	p-value	R^2_m	AIC
log10(V) ~ U	Intercept	-8.9310	0.367	-24.32	0.00		
	U	0.2711333	0.097	2.76	0.008	0.18	87.98
log10(V) ~ log10 (ε)	Intercept	-4.54	1.129	-4.02	0.000		
	log10 (ε)	0.541	0.175	3.08	0.004	0.11	84.4
log10(V) ~ P_A	Intercept	-9.307	0.638	-14.587	0.000		
	P_A	0.0002	0.0001	2.275	0.032	0.18	67.09
log10(V) ~ U + P_A	Intercept	-9.848	0.858	-11.469	0.000		
	U	0.198	0.215	0.923	0.36		
	P_A	2.04	1.10	1.85	0.076	0.20	68.24
log10(V) ~ U + log10 (ε)	Intercept	-5.76	1.76	-3.26	0.002		
	U	0.139	0.157	0.888	0.381		
	log10 (ε)	0.426	0.216	1.97	0.057	0.259	79.31

Table S4b: Effects of physical and chemical parameters on the microbubble void fraction shown by linear model. The predictors include hourly average wind speed (U), the TKE dissipation rate (ε), and the particle abundance (P_A). R^2 is coefficient of determination.

Predicted	Predictor	Estimate	Std. Error	t-value	p-value	R^2
log10(V)	Intercept	-8.606	0.418	-20.576	0.00	
	U	0.137	0.120	1.139	0.273	0.08
	Intercept	-6.381	1.294	-4.931	0.000	
	log10 (ε)	0.274	0.196	1.399	0.184	0.13
	Intercept	-7.475	1.1482	-6.5111	0.0003	
	P_A	-0.0001	0.0002	-0.4996	0.6327	0.034

Table S5: Summary of the linear mixed-effects model evaluating the effects of transect type and water depth on individual microbubble void fraction measurements. Sampling location and day were included as a random intercept.

LME Model	Fixed effect	Estimate	Std. Error	t value	p-value
Log10(V) ~ Transect + Depth	Intercept	-7.97131	0.467	-17.059	<0.001
	Transect (NonReed vs Reed)	-0.828	0.647	-1.281	0.269
	Depth (m)	-0.138	0.057	-2.445	0.037

References

(1) Wu, X.; Chahine, G. L. Development of an Acoustic Instrument for Bubble Size Distribution Measurement. *J. Hydrodyn.* **2010**, *22* (1), 325–331.

(2) Rehman, F.; Medley, G. J. D.; Bandulasena, H.; Zimmerman, W. B. J. Fluidic Oscillator-Mediated Microbubble Generation to Provide Cost Effective Mass Transfer and Mixing Efficiency to the Wastewater Treatment Plants. *Environ. Res.* **2015**, *137*, 32–39.

(3) Young, D. L.; McFall, B. C.; Bryant, D. B. Bubble Image Velocimetry with a Field-Deployable Acoustic Camera. *Meas. Sci. Technol.* **2018**, *29* (12), 125302.

(4) Wu, X. J.; Chahine, G. L. Development of an Acoustic Instrument for Bubble Size Distribution Measurement. *J. Hydrodyn.* **2010**, *22* (5 SUPPL. 1), 330–336. https://doi.org/10.1016/S1001-6058(09)60214-6.

(5) Thorpe, S. A. On the Clouds of Bubbles Formed by Breaking Wind-Waves in Deep Water, and Their Role in Air-Sea Gas Transfer. *Philos. Trans. R. Soc. London, Ser. A Math. Phys. Sci.* **1982**, *304* (1483), 155–210. https://doi.org/10.1098/rsta.1982.0011.

(6) Medwin, H. In Situ Acoustic Measurements of Microbubbles at Sea. *J. Geophys. Res.* **1977**, *82* (6), 971–976.

(7) Wanninkhof, R. Relationship between Wind Speed and Gas Exchange over the Ocean Revisited. *Limnol. Oceanogr. Methods* **2014**, *12* (JUN), 351–362. https://doi.org/10.4319/lom.2014.12.351.

(8) Battino, R. The Ostwald Coefficient of Gas Solubility. *Fluid Phase Equilib.* **1984**, *15* (3), 231–240.

(9) Asher, W. E.; Wanninkhof, R. The Effect of Bubble-mediated Gas Transfer on Purposeful Dual-gaseous Tracer Experiments. *J. Geophys. Res. Ocean.* **1998**, *103* (C5), 10555–10560.

(10) Gelbrecht, J.; Fait, M.; Dittrich, M.; Steinberg, C. Use of GC and Equilibrium Calculations of CO2 Saturation Index to Indicate Whether Freshwater Bodies in North-Eastern Germany Are Net Sources or Sinks for Atmospheric CO2. *Fresenius. J. Anal. Chem.* **1998**, *361*, 47–53.

(11) Xiao, S.; Liu, L.; Wang, W.; Lorke, A.; Woodhouse, J.; Grossart, H.-P. A Fast-Response Automated Gas Equilibrator (FaRAGE) for Continuous in Situ Measurement of CH 4 and CO 2 Dissolved in Water. *Hydrol. Earth Syst. Sci.* **2020**, *24* (7), 3871–3880.

(12) Wiles, P. J.; Rippeth, T. P.; Simpson, J. H.; Hendricks, P. J. A Novel Technique for Measuring the Rate of Turbulent Dissipation in the Marine Environment. *Geophys. Res. Lett.* **2006**, *33* (21), 21608. https://doi.org/10.1029/2006GL027050.

(13) Monin, A.; Yaglom, A. *Statistical Fluid Mechanics, Volume II: Mechanics of Turbulence*; 2013.

(14) Bluteau, C. E.; Jones, N. L.; Ivey, G. N. Estimating Turbulent Kinetic Energy Dissipation Using the Inertial Subrange Method in Environmental Flows. *Limnol. Oceanogr. Methods* **2011**, *9* (JULY), 302–321. https://doi.org/10.4319/lom.2011.9.302.

(15) Borchers, H. W. Pracma: Practical Numerical Math Functions. *R Packag. version* **2019**, *2* (9), 519.

(16) Hofmann, H.; Lorke, A.; Peeters, F. The Relative Importance of Wind and Ship Waves in the Littoral Zone of a Large Lake. *Limnol. Oceanogr.* **2008**, *53* (1), 368–380. https://doi.org/10.4319/LO.2008.53.1.0368.

(17) Vachon, D.; Prairie, Y. T.; Cole, J. J. The Relationship between Near-surface Turbulence and Gas Transfer Velocity in Freshwater Systems and Its Implications for Floating Chamber

Measurements of Gas. *Wiley Online Libr. Vachon, YT Prairie, JJ ColeLimnology Oceanogr. 2010•Wiley Online Libr.* **2010**, *55* (4), 1723–1732. https://doi.org/10.4319/LO.2010.55.4.1723.

(18) MacIntyre, S.; Crowe, A. T.; Cortés, A.; Arneborg, L. Turbulence in a Small Arctic Pond. *Limnol. Oceanogr.* **2018**, *63* (6), 2337–2358. https://doi.org/10.1002/LNO.10941.

(19) Brissette, F. P.; Tsanis, I. K.; Wu, J. Wave Directional Spectra and Wave-Current Interaction in Lake St. Clair. *J. Great Lakes Res.* **1993**, *19* (3), 553–568. https://doi.org/10.1016/S0380-1330(93)71241-1.

(20) Young, I. R.; Verhagen, L. A.; Banner, M. L. A Note on the Bimodal Directional Spreading of Fetch-Limited Wind Waves. *J. Geophys. Res.* **1995**, *100* (C1), 773–778. https://doi.org/10.1029/94JC02218.

(21) Mirshak, R.; Kelley, D. E. Inferring Propagation Direction of Nonlinear Internal Waves in a Vertically Sheared Background Flow. *J. Atmos. Ocean. Technol.* **2009**, *26* (3), 615–625. https://doi.org/10.1175/2008JTECHO632.1.

(22) Foken, T. Micrometeorology. *Micrometeorology* **2008**, 1–306. https://doi.org/10.1007/978-3-540-74666-9/COVER.

(23) Forristall, G. Z.; Ewans, K. C. Worldwide Measurements of Directional Wave Spreading. *J. Atmos. Ocean. Technol.* **1998**, *15* (2), 440–469. https://doi.org/10.1175/1520-0426(1998)015<0440:WMODWS>2.0.CO;2.

(24) Muggeo, V. M. R. Segmented: An R Package to Fit Regression Models with Broken-Line Relationships. *R news* **2008**, *8* (1), 20–25.

(25) Taylor, G. I. Statistical Theory of Turbulenc. *Proc. R. Soc. London. A. Math. Phys. Sci.* **1935**, *151* (873), 421–444. https://doi.org/10.1098/rspa.1935.0158.

(26) Vassilicos, J. C. Dissipation in Turbulent Flows. *Annu. Rev. Fluid Mech.* **2015**, *47* (Volume 47, 2015), 95–114. https://doi.org/10.1146/annurev-fluid-010814-014637.

(27) Woolf, D. K. Bubbles and the Air-sea Transfer Velocity of Gases. *Atmos. - Ocean* **1993**, *31* (4), 517–540. https://doi.org/10.1080/07055900.1993.9649484.

(28) Hill, K. D.; Dauphinee, T. M.; Woods, D. J. The Extension of the Practical Salinity Scale 1978 to Low Salinities. *IEEE J. Ocean. Eng.* **1986**, *11* (1), 109–112. https://doi.org/10.1109/JOE.1986.1145154.

(29) Memery, L.; Merlivat, L. Modelling of Gas Flux through Bubbles at the Air-Water Interface. *Tellus B Chem. Phys. Meteorol.* **1985**, *37* (4–5), 272. https://doi.org/10.3402/TELLUSB.V37I4-5.15030.

(30) Mays, L. W. *Water Resources Engineering*; John Wiley & Sons, 2010.

(31) Chen, C. T.; Millero, F. J. The Use and Misuse of Pure Water PVT Properties for Lake Waters. *Nat. 1977 2665604* **1977**, *266* (5604), 707–708. https://doi.org/10.1038/266707a0.

(32) Leifer, I.; Patro, R. K. The Bubble Mechanism for Methane Transport from the Shallow Sea Bed to the Surface: A Review and Sensitivity Study. *Cont. Shelf Res.* **2002**, *22* (16), 2409–2428. https://doi.org/10.1016/S0278-4343(02)00065-1.

(33) Mcginnis, D. F.; Kirillin, G.; Tang, K. W.; Flury, S.; Bodmer, P.; Engelhardt, C.; Casper, P.; Grossart, H. Enhancing Surface Methane Fluxes from an Oligotrophic Lake: Exploring the Microbubble Hypothesis. **2015**. https://doi.org/10.1021/es503385d.

(34) Deane, G. B.; Stokes, M. D. Scale Dependence of Bubble Creation Mechanisms in Breaking Waves. *Nature* **2002**, *418* (6900), 839–844. https://doi.org/10.1038/nature00967.

(35) Phelps, A. D.; Ramble, D. G.; Leighton, T. G. The Use of a Combination Frequency Technique to Measure the Surf Zone Bubble Population. *J. Acoust. Soc. Am.* **1997**, *101* (4), 1981–1989. https://doi.org/10.1121/1.418199.

(36) Trevorrow, M. V. Multifrequency Acoustic Investigations of Juvenile and Adult Fish in Lake Biwa, Japan. *J. Acoust. Soc. Am.* **1996**, *100* (5), 3042–3052. https://doi.org/10.1121/1.417116.

(37) Ling, S. C.; Pao, H. P. Study of Micro-Bubbles in the North Sea. *Sea Surf. Sound* **1988**, 197–210. https://doi.org/10.1007/978-94-009-3017-9_15.

(38) Randolph, K.; Dierssen, H. M.; Twardowski, M.; Cifuentes-Lorenzen, A.; Zappa, C. J. Optical Measurements of Small Deeply Penetrating Bubble Populations Generated by Breaking Waves in the Southern Ocean. *J. Geophys. Res. Ocean.* **2014**, *119* (2), 757–776. https://doi.org/10.1002/2013JC009227;SUBPAGE:STRING:FULL.

(39) Oakey, N. S. Determination of the Rate of Dissipation of Turbulent Energy from Simultaneous Temperature and Velocity Shear Microstructure Measurements. *J. Phys. Oceanogr.* **1982**, *12* (3), 256–271. https://doi.org/10.1175/1520-0485(1982)012.
